# Is learning a logographic script easier than reading an alphabetic script for German children with dyslexia?

**DOI:** 10.1371/journal.pone.0282200

**Published:** 2023-02-24

**Authors:** Stephan Kuester-Gruber, Theda Faisst, Vera Schick, Giulia Righetti, Christoph Braun, Angelika Cordey-Henke, Matthias Klosinski, Ching-Chu Sun, Susanne Trauzettel-Klosinski

**Affiliations:** 1 Vision Rehabilitation Research Unit, Center for Ophthalmology, University of Tuebingen, Tuebingen, Germany; 2 China Center Tuebingen, Erich Paulun Institute, University of Tuebingen, Tuebingen, Germany; 3 MEG-Center, University of Tuebingen, Tuebingen, Germany; 4 Hertie Institute for Clinical Brain Research, University of Tuebingen, Tuebingen, Germany; 5 DiPSCO, Department of Psychology and Cognitive Science, University of Trento, Rovereto, Italy; 6 Center for Mind/Brain Sciences, University of Trento, Rovereto, Italy; 7 Department of Child and Adolescent Psychiatry and Psychotherapy, University Hospital Munich, LMU Munich, Muenchen, Germany; 8 Department of General Linguistics, University of Tuebingen, Tuebingen, Germany; Public Library of Science, UNITED STATES

## Abstract

**Purpose:**

Developmental dyslexia in alphabetic languages (DD) is characterized by a phonological deficit. Since logographic scripts rely predominantly on visual and morphological processing, reading performance in DD can be assumed to be less impaired when reading logographic scripts.

**Methods:**

40 German-speaking children (18 with DD, 22 not reading-impaired—group C; 9–11 years) received Chinese lessons. Eye movements (EM) were recorded during naming single alphabetic words, pictures (confrontational) and Chinese characters to be named in German and Chinese. The main outcome variables were: Articulation latency, numbers and durations of fixations. Quality of life (QoL) was assessed by questionnaires.

**Results:**

While reading alphabetic words, articulation latencies and numbers of fixations were significantly higher for group DD than for group C (AL-DD = 1.13, AL-C = 0.84, p< .001; FN-DD = 3.50; FN-C = 2.00, p< .001). For naming pictures and Chinese characters in German and in Chinese, no significant group differences were found for any of the EM variables. The percentage of correct answers was high for German naming (DD = 86.67%, C = 95.24%; p = .015) and lower for Chinese naming in both groups, but significantly lower in group DD, especially for Chinese naming (DD = 56.67%, C: 83.77%; p = .003). QoL differed between groups from the children’s perspective only at posttest. Parents of group DD perceived their children`s QoL to be lower compared with parents of group C at pre- and posttest.

**Conclusions:**

Children with dyslexia performed as well as group C during naming Chinese characters in German and in Chinese regarding their EM variables, presumably because they processed Chinese characters by the visuo-spatial pathway with direct access to the semantic system. However, the significantly lower percentage of correct answers especially during Chinese naming showed that group DD had more difficulties naming Chinese characters than group C, which could be attributed to their phonological deficit, among other factors.

**Trial registration:**

German clinical trials register (DRKS00015697).

## Introduction

Developmental dyslexia (DD) is a multicausal disorder that specifically affects learning to read and write and that cannot be accounted for by reduced intelligence, insufficient education or sensory or neurological diseases [1]. The prevalence ranges from 4–6% in German-speaking children [2, 3]. An even higher prevalence has been reported in non-regular languages such as English [4]. Regarding alphabetic languages, it is generally agreed that DD is a language processing disorder characterized predominantly by a phonological deficit.

Phonological deficits can be caused by diminished phonological awareness, e.g. by insufficient identification, analysis, synthesis and manipulation of language units on a sublexical level, where grapheme-phoneme conversions are used, especially in regular orthographies [5–11].

Another important aspect of phonological information processing is phonological working memory (the phonological loop) where storing and processing of language information need to be performed simultaneously. There are two subunits of the phonological loop: The phonological buffer, which is responsible for short-term storage, and the phonological rehearsal for actively maintaining language information, both of which can be impaired in children with dyslexia [12–15].

A third process plays a role for phonological information processing: Rapid Automatized Naming (RAN), which requires fast and automatized access to phonological representations of visually presented stimuli. RAN is based on the serial (repeated) presentation of pictures, colors or digits to be named as quickly as possible. Deficits in RAN are mainly attributed to a deficit in rapid processing of visual stimuli [16, 17]. RAN deficits are often, but not consistently, associated with DD and have been described in numerous studies with contradictory explanations [16–28].

The working memory model of Baddeley [12–14] contains not only the above-mentioned phonological loop, but also a visual subunit, the visuo-spatial sketchpad. According to some studies [29, 30], impairments of visual processing do not play an essential role in DD in alphabetic languages.

Discrete visual dysfunctions have been reported, such as enhanced crowding [31–36], a magnocellular deficit [37, 38], or a reduced visual attention span [39–41]. However, these findings remain controversial and have been questioned by authors of recent publications [20, 36, 42], who have hypothesized that a visual deficit may only be present in subgroups with DD.

We have examined the performance of children with DD during a task that did not require grapheme-phoneme conversion, where the primary process is visuo-spatial, but not letter-mediated by presenting pictures of familiar objects that were grouped like words and displayed in a line like in a text [30].

The task was to name the pictures without time pressure and repetitions, which is referred to as “confrontational naming” (CN) (in contrast to RAN). In this task, the children with DD performed as well as their age-matched not reading-impaired control group, which we have shown in studies using eye movement recording and magneto-encephalography [30, 43]. Other studies have shown confirming and contradictory results using different approaches (see Discussion) [44, 45].

The unimpaired performance of children with DD in CN tasks indicates neither a visual deficit regarding the discrimination of stimuli, nor a primary oculomotor dysfunction [30].

These results raised the question, whether these children might learn a non-alphabetic, logographic script more easily than an alphabetic one. Therefore, we investigated whether children with dyslexia in an alphabetic language could learn a non-alphabetic language as well as not reading-impaired, age-matched children.

Several studies have reported that reading Chinese requires different skills than reading an alphabetic script [46–48]. In contrast to reading an alphabetic language, there is a less consistent correlation between phonological awareness and dyslexia in Chinese [49–52]. In the process of learning Chinese at an early age (below second grade), visual skills seem to predict successful reading, which suggests a contribution by a logographic/visual phase [48, 53]. Besides pure visual memory, further skills such as speed of visual processing and visual perception [53], as well as visuo-spatial abilities [54], are important at initial stages of learning Chinese. In addition, many studies refer to morphological awareness as a major component of reading Chinese characters [49, 51, 55–61]. A morpheme is the smallest meaningful or a consistently occurring element in a language. Morphological awareness refers to the capacity to reflect on and to manipulate morphemes and the morphological structure of words.

Other authors emphasize the importance of copying Chinese characters when Chinese children learn to read, which stresses visual similarities and differences [62, 63]. In addition, learners of Chinese focus mainly upon the meaning of characters or radicals, while their sound is rarely mentioned [64]. Likewise, Zhou et al. [47] observed that Chinese learners are more likely to rely upon semantics than those who learn alphabetic languages.

Previous studies on reading Chinese focused on reading two- or more-character words and sentences mostly in not reading-impaired native speakers of Chinese. In this condition, eye movements are influenced by various factors such as word length, artificial word spacing, parafoveal processing and font size [65–70]. In contrast, the current study uses only single-character Chinese words in order to focus on eye movement patterns in children with dyslexia with respect to phonological versus visual processing, which is not directly comparable to reading Chinese sentences.

A study of 9 children who were severely impaired in alphabetical reading reported that they were capable of learning Chinese characters when naming them in their native English [71]. However, that study did not require naming the words in Chinese as we required in our study. In the present study, we examined the performance of children with dyslexia in a regular orthography and an age-matched, not reading-impaired control group with German as primary language, who were asked to name Chinese characters in German as well as in Chinese.

The aims of our study were to investigate:

1. Whether the phonological deficit in the alphabetic system plays a role in Chinese reading or whether the children with DD can learn Chinese characters more easily than alphabetic words.
2. Whether they perform as well during Chinese naming, but worse in alphabetic naming when compared with the not reading-impaired control group.
3. Whether performance in confrontational picture naming might be a predictor of the ability to read Chinese characters.

To address these questions, we examined reading and naming during four different tasks: Reading alphabetic words, naming pictures (confrontational), naming Chinese characters in German and in Chinese.

Furthermore, we studied the eye movement patterns during these four tasks, because eye movement recordings can quantify reading performance and naming difficulties. This is achieved by examining variables such as number of fixations, fixation duration, and articulation latency (by simultaneous recording of speech), all of which reflect the phonological deficit in alphabetic reading [72–75].

In addition, we applied questionnaires to appraise subjective problems and quality of life (QoL). With regard to psychological effects, it has been shown that DD diminishes QoL [76]. Children with DD often face stigmatization and are more likely to struggle with secondary social and psychological problems [77]. Experiences connected with DD can affect their self-esteem [78] and render them at a higher risk for anxiety and depression [79]. In contrast, children with dyslexia could be given a chance to enhance their self-esteem by experiencing successful learning of a logographic script. In the past, primary foci of research on DD were the deficits of these children, while their potential strengths, such as visual abilities, were rarely considered. If reading Chinese characters relied on different skills than alphabetic reading, it would indicate areas of competence in children with dyslexia that could be utilized to support them in everyday demands by challenging their visual abilities more often. This could provide a competitive advantage at schools teaching Chinese and in their future professional life and could prevent secondary social and psychological disorders [77].

We would like to emphasize that using a block of Chinese lessons as an intervention was not meant to suggest that this could improve dyslexia. Rather, the lessons were used to examine the visuo-spatial abilities of these children and to explore the possibility to support these abilities by learning a logographic language. We are aware of the fact that children with dyslexia need specific treatment in the alphabetic language.

Whereas most of previous research on alphabetic languages has been based on English (with its irregular orthography), only one study compared DD in a non-alphabetic language (Chinese) with a regular alphabetic language (Spanish) [80]. In that study, sensory cues-to-speech rhythms (rise time sensitivity) were assessed in native speakers of Spanish, Chinese, and English with dyslexia.

It was the aim of our study to look for agreements and differences between children with and without dyslexia by recording eye movements during different tasks that are characterized by different visual and phonological demands. Below we list our hypotheses and assign specific procedures to test them:

Hypothesis 1: Children with dyslexia will show a **different eye movement pattern** (more fixations, longer articulation latencies) when reading **alphabetic words** with significantly worse performance than the children without dyslexia. To answer this question, we examined the eye movement variables: Number of fixations, fixation durations, and articulation latencies.

Hypothesis 2: Children with dyslexia will perform **as well as those without when naming pictures** in a confrontational naming task. Again, we tested this by examining the eye movement variables and the error rate.

Hypothesis 3: Group DD will be able to **learn Chinese characters as well** as the children without dyslexia. To answer this question, we examined their eye movement variables and their error rate.

Hypothesis 4: The children in group DD and their parents will report a **lower QoL** than those without dyslexia. To answer this question, we applied a QoL questionnaire.

**The new aspects** of our present study include: 1. Eye movement measurements during reading alphabetic words aloud in order to assess the phonological deficit, CN-naming of pictures to test visuo-spatial processing, and naming Chinese characters in German and in Chinese, 2. Children with dyslexia in a regular orthography (German) learned Chinese characters and their performance was compared with that of an age-matched control group, 3. QoL before and after a block of Chinese lessons. Furthermore, we were interested in understanding the children’s experience of learning a new language with a different approach than their native language and its potentially positive effect on their QoL.

## Methods

### Study design

**Fig 1:**
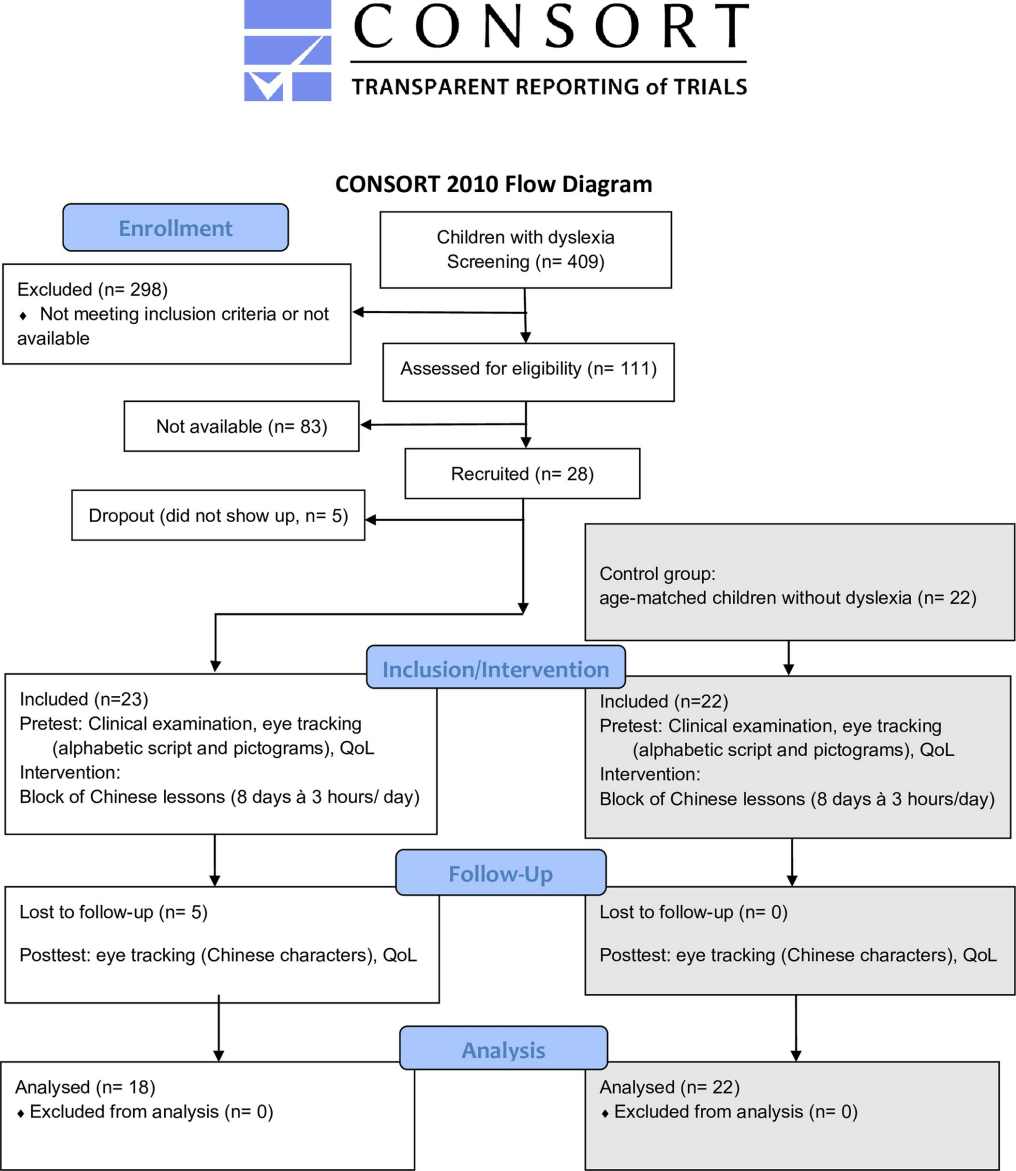
CONSORT flow diagram of the study. For details see the following text.

The CONSORT flow diagram of the study is shown in Fig 1.

The following examinations were performed during the first visit to our eye hospital (pretest): Ophthalmological examinations, quality of life (QoL) questionnaire, reading tests, eye tracking during reading alphabetical words and naming pictures of objects. During the following school holidays, the children participated in a block of Chinese lessons (8 days, 3 hours per day) at the China Center Tuebingen (CCT). At the second visit to the hospital (posttest), eye tracking during naming single Chinese characters was performed and QoL was assessed again.

### Participants

The children with dyslexia (group DD) were recruited mainly by the Department of Child and Adolescent Psychiatry and Psychotherapy of the University of Tuebingen, which was part of a collaborative effort. Additional children with dyslexia were recruited by newsletter, newspaper advertisement, or by local child psychiatrists, local schools, and personal contacts with professionals. We screened 409 children with dyslexia, but 298 had to be excluded because they did not meet the inclusion criteria due to additional attention deficits (140), or other comorbidities. On the other hand, this enabled us to work with a homogeneous group of children with dyslexia without additional pathologies. In addition, some children could not participate, because they lived too far away to travel 10 times to Tuebingen (two visits to the eye hospital and eight days for the lessons) (Fig 1). Therefore, only 111 children were eligible to participate. Unfortunately, 83 of them did not answer our invitation, probably because the children had no time to participate because of too many other scheduled activities or due to change of address, so that they might not have received our letter. The remaining 28 children with dyslexia were recruited, and after 5 children did not show up, finally 23 children with dyslexia could be included in the study. Five of these were lost during follow-up, because they missed more than 2 out of 8 lessons. Therefore, all steps of the data analysis were performed on the data from 18 children with dyslexia (14 boys, 4 girls; mean age 10 years, 2.5 months; SD 11 months). The diagnosis of dyslexia was made by experts in the field, i.e. child psychiatrists and specialized psychologists based on standardized test procedures that are generally used and recommended by professional societies in the field–for details see below.

The 22 members of the control group (group C) were healthy, not reading-impaired, age-matched children of 4^th^ and 5^th^ grade (i.e. 9–11 years), and were recruited by newsletter, newspaper advertisement, local schools and personal contacts (10 boys, 12 girls; mean age 10 yrs, 2 months; SD 9 months).

Based on one of our own studies on reading alphabetic words [43], a necessary sample size of 12 children (6 in each group) was calculated (for details see section “Statistical Methods”). Thus, the 18 children with and the 22 children without dyslexia in the present study clearly exceed the required sample size.

It was our goal to study whether learning Chinese is a strategy for individual dyslexics to escape their reading disability. To develop a meaningful strategy, we would expect that it should be effective in single cases rather than for groups with several tens of participants.

Recruitment, pretest and posttest were performed from October 2018 to November 2019.

### Participant selection

Inclusion criteria:

- Developmental dyslexia (ICD-10 F81.0), diagnosed by child and adolescent psychiatrists based on standardized test procedures (see below) according to the definition of the WHO (2008) [1] and the guidelines of the German Association for Child and Adolescent Psychiatry, Psychosomatics and Psychotherapy (Deutsche Gesellschaft für Kinder und Jugendpsychiatrie, Psychosomatik und Psychotherapie e.V. (reported in English by Galuschka and Schulte-Körne 2016) [81], for details see below.
- 4^th^ to 5^th^ grade (9–11 years old), at inclusion in the study, see Table 1.
- German as first language
- Healthy control children without reading and writing problems

Exclusion criteria:

- Reading impairment of other origin
- Any disease of the eyes and visual pathway
- Comorbidities such as ADHS, ADS, emotional disorders

**Table 1:** Data at baseline.

id	group	age [years]	sex	reading speed (ZLT) [wpm]	reading errors	va	va crowding	crowding difference [log]	school grade
1	DD	10.4	g	73.26	12	1.4	1	2	5
2	DD	9.7	b	50	8	1.4	1.25	1	4
3	DD	11.3	b	54	8	1.4	1	2	5
4	DD	10.8	b	78	1	1.4	1.2	1	5
5	DD	10.1	b	71	9	1.25			4
6	DD	12.6	b	61	4	1.4	0.9	2.25	6
7	DD	10.1	g	39	2	1.4	1.4	0	4
8	DD	11.0	b	118.5	2	1.4	1	2	5
9	DD	10.9	b	21.4	26	1.4	1	2	5
10	DD	9.4	g	45.8	2	1.4	0.9	2.25	4
11	DD	11.2	b	56	0	1.25	0.7	2.5	5
12	DD	11.9	b	25	20	1.4	0.9	2.25	5
13	DD	11.5	b	107	8	1.4	0.9	2.25	5
14	DD	9.6	b	57	2	1.25	1	1	4
15	DD	10.1	b	79	4	1.4	1	2	4
16	DD	9.7	b	58	3	1.4	1	2	4
17	DD	10.1	g	44	3	1.25	0.9	1.25	4
18	DD	9.3	b	90.7	4	1.4	1	2	4
19	C	9.2	b	111	0	1.4	1	2	4
20	C	10.6	b	136	0	1.4	1.4	0	5
21	C	9.0	g	63.5	0	1.25	1	1	4
22	C	11.5	g	141	0	1.4	1	2	5
23	C	10.2	g	123.8	0	1.4	1	2	5
24	C	10.9	b	149	0	1.4	1.25	1	5
25	C	10.2	b	106	0	1.4	1.4	0	4
26	C	10.2	b	116	0	1.4	1	2	4
27	C	11.0	g	105	0	1.4	1.25	1	5
28	C	11.0	g	132.6	0	1.4	1	2	5
29	C	9.4	g	119	0	1	0.6	2	4
30	C	9.6	b	110	0	1.4	1	2	4
31	C	9.9	b	136	0	1.4	1.4	0	4
32	C	9.9	g	138	0	1.4	1.25	1	4
33	C	11.6	g	139	0	1.4	1.4	0	5
34	C	10.4	b	141	0	1.4	1	2	4
35	C	9.6	g	111.3	0	1.4	1.4	0	4
36	C	10.1	b	132	0	1	0.8	1	4
37	C	10.7	g	150	0	1	0.8	1	5
38	C	11.1	g	118	0	1.25	0.9	1.25	5
39	C	10.8	g	163	0	1.4	1.4	0	5
40	C	9.4	b	123.8	0	1.4	1.25	1	4

DD: children with developmental dyslexia (group DD), C: control (group C), g: girl, b: boy, ZLT: Zürcher reading Test (ZLT II), wpm: words per minute, va: visual acuity (decimal)

The standardized diagnosis of dyslexia consists of various tests. Their application in the individual case depends on the age, school type, school grade etc.

The test battery always contains an intelligence test, a reading test AND a spelling test, or a combined reading and spelling test. Our external co-operation partners applied the following tests in all the children before they were included in our study:

- an **intelligence test** (e.g. HAWIK-IV, Wechsler Intelligence test for children WISC-IV, CFT-20-R),
- **a reading test** (e.g. WRT 4/4, WRT 4/5, WRT 6, Zürcher Lesetest ZLT, Würzburger Leise Leseprobe WLLP, Salzburger Lese-Screening SLS 2–9 B1, DRT 3 A)
- **a spelling tests** (e.g. Hamburger Schreibprobe HSP 5B, Würzburger Rechtschreibtest)
- **a combined reading and spelling test** (e.g. Salzburger Lese-Rechtschreibtest SLRT I+II)
- **additional tests** depending on the child`s history: Standardized questionnaires regarding quality of life and reports by parents.

Detailed descriptions are shown in the guidelines of the German Association for Child and Adolescent Psychiatry, Psychosomatics and Psychotherapy (reported in English by Galuschka and Schulte -Koerne 2016) [81].

The data of all participants at baseline can be found in Table 1.

The primary purpose of the study was to assess the difference between children with dyslexia and a control group regarding the 4 different tasks (reading alphabetic words, naming pictures, naming Chinese characters in German, and naming Chinese characters in Chinese). For this reason, the primary outcome variables were the eye movements (articulation latencies, number of fixations and fixation durations) assessed by eye tracking. Secondary outcome variables were the resulting difficulties based on subjective reports by the children and their parents.

The project was approved by the ethics committee of the University of Tuebingen medical faculty, and informed written consent was obtained from the parents and children. The research adhered to the tenets of the Declaration of Helsinki. The study was reported in an open-source online registry as a non-randomized interventional study (No. DRKS00015697), German Clinical Trials Register, Cologne, Germany. The term “intervention” is used for any experimental intervention, in contrast to observational or epidemiological studies.

### Examinations

Neuro-ophthalmological examinations were performed to exclude an ophthalmological cause for the reading difficulty. These included: Distance and near visual acuity, accommodation, refraction, crowding (the difference of near visual acuity between single optotypes and grouped optotypes—assessed by the Oculus near vision charts, Landolt rings), eye position, strabismus, motility, convergence, stereopsis, saccades, eye dominance and pupil reaction, and morphology of the outer and inner segments of the eye.

We used the Zürcher reading Test (ZLT II) [82], for measuring reading performance and speed, because it provides standardized and evaluated reading texts for each class from second to sixth grade. We used sentences for the 4^th^ and 5^th^ grade. In the test, reading time and reading errors were recorded using word lists and texts of varying difficulty. Reading speed in words per minute (wpm) was calculated as follows: Reading time in seconds divided by the sum of correctly read words multiplied by 60.

### Eye tracking

#### Instrumentation and experimental setup

We used two eye tracking systems, because they supplement each other favorably:

1. **Infrared Eye Tracker** (IR-ET), high temporal resolution and automated measurement of eye movement variables.
2. **Scanning Laser Ophthalmoscope** (SLO), simultaneous visualization of retina and stimulus.

Both methods allow voice recording, e.g. verbal naming of stimuli, which facilitates offline analysis of naming errors from the soundtrack.

Both methods are described in detail below:

*Infrared Eye Tracker (IR-ET)*. The infrared eye tracking device (JAZZ-novo, Ober Consulting, Poznan, Poland) was used to monitor eye movements for the detailed analysis of the difficulties during the 4 naming tasks. The device has a spatial resolution of 0.1 degrees and a sampling rate of 1000 Hz. Correcting glasses can be placed directly on the apparatus and do not interfere with the eye movement signal. The IR-ET device is especially suited for children because of the easy set-up and minimal intrusiveness. The children’s voices were recorded with the additional JAZZ-novo audio signal recording. The soundtrack allows measuring the articulation latency and monitoring the correctness of the answers. The stimuli were presented on a 21-inch CRT monitor at 25 cm distance, while a chin rest limited head movements. We recorded the voice, as well as the movements during the whole experiment.

Main outcome variables were:

1. Articulation latency as a measure of naming speed. This was defined as the time between the end of the first saccade to the stimulus and the beginning of articulation (indicated by the visible signal in the audio track).
2. Number of fixations to assess the difficulty during the child`s analysis of the stimulus. A fixation was defined as a period without eye movements of = > 0.5° that was followed or preceded by a saccade and was at least 100 ms long.
3. Fixation duration was used as a measure of information acquisition time (processing time). A saccade was defined as a fast eye movement of minimal amplitude of 0.5° (the width of the letter “n”). The IR-ET defined additional saccade characteristics by using internal software (minimal velocity 20 deg/s, minimal peak velocity 75 deg/s, minimal acceleration rate 1000 deg/s^2^). Individual fixation duration per item was calculated (neglecting saccade duration) as

$$fixation\;duration\;[s]\;=\;\frac{articulation\;latency\;[s]}{number\;of\;fixations}$$

Before starting the examination, the children performed a short calibration procedure by making saccades between four crosses in the corners of the screen, which were to be fixated in a clockwise order. Before each stimulus was shown, the children fixated a cross (size = 0.8°) that was presented 5° left of the center of the screen. Then the stimulus appeared in the center to trigger a saccade to the stimulus with a defined landing position.

#### Eye tracking by SLO

**Fig 2:**
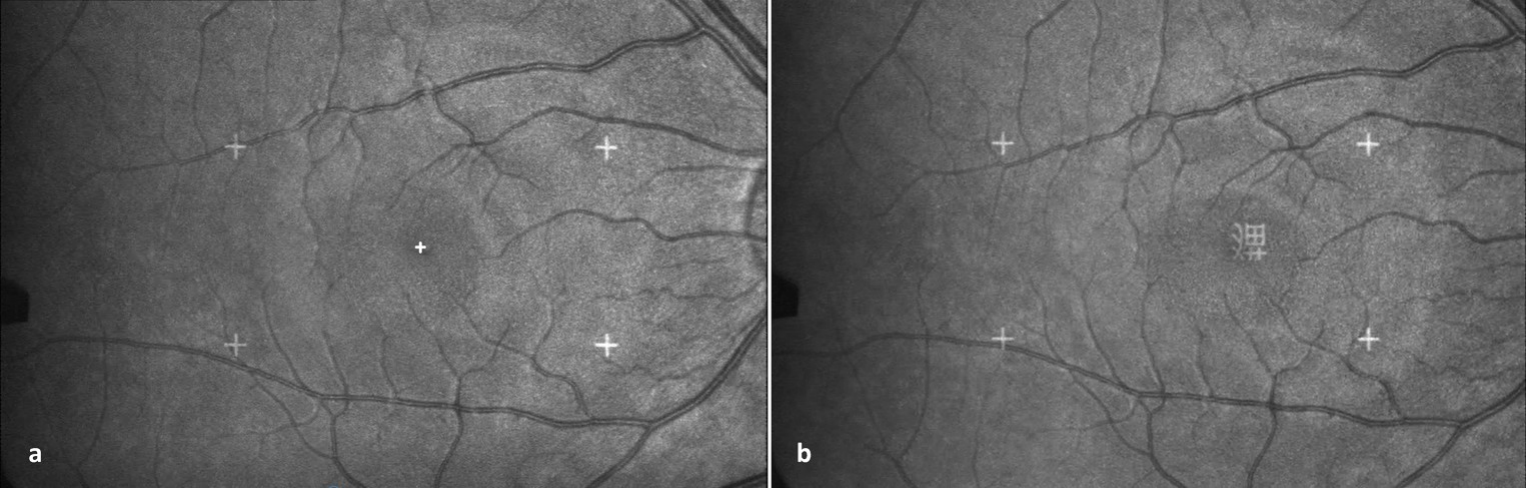
Analysis of the SLO data. The retina and the stimulus (here the character cat 猫) are seen upside down only by the examiner. The stimulus is seen upright by the child. The Chinese character is fixated with the fovea.

A Scanning Laser Ophthalmoscope (SLO 101, Rodenstock Instruments, Ottobrunn, Germany) was used to assess the fixation locations on the stimulus. During the examination, the SLO scans stimuli directly onto the retina, which is visible simultaneously with the stimuli on a video monitor (Fig 2). This shows the absolute position of the center of the fovea relative to the stimulus with a spatial resolution of 0.1 deg. The temporal resolution of the SLO is limited by its 25 Hz sampling rate (50 half-frames/s). The SLO examination was performed on the dominant eye. Eye dominance was determined by the pinhole test [83]. The video signal could be recorded for further analysis by image processing software.

A total of 58,694 half-frames (eye coordinates) were included in our analysis, which was a very time-consuming procedure. In order to determine the fixation location, we used custom-designed image analysis software that tracks the position of a user-defined landmark, e.g. a retinal vessel branching. This detects retinal movements and allows determining the coordinates of the fovea relative to the image of the stimulus, both of which are visible on the image of the retina (Fig 2). The definition of the eye movement variables was the same as for the IR-ET method.

After separate analysis of the eye movements by both methods, we found good agreement between the data and therefore, we combined them.

### Single stimuli

The present study focused on learning and naming Chinese characters. However, there were two reasons why we also included examinations of pictures and alphabetic words: Firstly, as verification test of our previous studies [30, 43, 72, 73], where we highlighted difficulties in reading alphabetic words and the unimpaired confrontational naming of pictures based on a higher number of stimuli. Secondly, pictures and alphabetic words were here also used as a control condition for the new stimuli (Chinese characters). See all stimuli in S1 File.

#### Reading single alphabetic words

The 16 alphabetic words (w1-w16) of different phonological difficulty based on length (short: 4 to 5 letters /long: 7 to 12 letters) and frequency in German (high-frequency words: absolute frequency in the basic vocabulary >50; low-frequency words: absolute frequency <3) were presented consecutively. The words were taken from the basic vocabulary for elementary schools [84]. Altogether, we had 4 different categories of linguistic difficulty: Short/frequent, short/rare; long/frequent, long/rare. They were set in Times New Roman at a font size of 14 pt, where a lower-case “n” was 0.5° wide, and were to be viewed at a distance of 25 cm.

#### Naming single pictures

The task was to name 6 single pictures of objects without time pressure (CN). The size of the pictures differed in horizontal and vertical extent between 1.8° and 4.4°. The size of the pictures was adapted to the size of the Chinese characters to match the aspect ratio of their templates. The main criteria for the selection of pictures were easy recognizability and familiarity of the depicted objects to children. An answer was considered correct if the participant could name the correct term or an “umbrella” term. For instance, for the stimulus picturing a “shark”, the answer “fish” was accepted as correct.

#### Naming single Chinese characters

The children were asked to name a total of 24 single Chinese characters that were selected from a storybook about the Chinese Zodiac. The one-character words were used as the basis for the lessons (see below). Twelve characters were to be named in German and 12 in Chinese. The characters were presented at the smallest font size that was still legible. In addition, typefaces in calligraphic styles were not adopted to avoid non-essential pixels and to provide simpler visualization of the characters.

The Chinese characters were set in the font SimHei (26 pt), which corresponded to 1.8° (width and height) at a viewing distance of 25 cm. The typeface and font size were used after consultation with native speakers from the China Center Tuebingen (CCT) and the Department of Linguistics at the University of Tuebingen. A dummy experiment was performed with two native speakers of Mandarin Chinese before the implementation of the experiment.

#### Error rates

The percentage of correct answers for each stimulus and task were verified by having the soundtrack analyzed by a Chinese teacher and a native speaker.

### Chinese lessons

The didactic concept and the materials were developed at the China Center Tuebingen (author VS). The Chinese lessons took place on eight consecutive days for three hours each; the teacher (author VS) was supported by a native Chinese speaker. The children with and without DD were taught together in small groups. The teachers did not know which of the children had been diagnosed with DD. Characteristics for the Chinese characters taught in class included visual complexity, degree of iconicity, composition and structure, similarity or dissimilarity, familiarity of the meaning and applicability in the lessons as well as phonetics. The stimuli were selected in cooperation with the Department of Linguistics. A total of 37 characters were taught during this period. An important didactic goal was to anchor the characters to be taught both in a context with culture-specific relevance and to embed them in authentic language interactions. The thematic frame of reference was the story of the origin of the Chinese zodiac based on the picture book *"Das kaiserliche Wettrennen”* (Original Title: The Imperial Race) [85]. Discovery-based learning, visual integrative approach, and cooperative learning were used. The teaching materials specially developed for the course promoted visual perception and memorization strategies. Both receptive and productive language skills were equally trained. To compensate for possible phonological deficits, the use of the alphabet was consistently avoided during the course: We used neither the romanization system Hanyu Pinyin (the phonemic transcription of the Chinese language) nor was the German meaning of the Chinese characters spelled in letters. The pronunciation of a character was judged to be correct if it was intelligible and if the initial and final sound was approximately correct (with a certain tolerance for difficult initials that do not exist in German). Since the participants were absolute beginners, the four tones were not included in the analysis. To support memorization and individual learning, the children were provided with a learning app specially created for the course contents. This learning app also did not contain any letters but showed a picture corresponding to the Chinese character regarding the meaning. The correct pronunciation of the Chinese character was played at button click on the character.

### Quality of life questionnaire for children and parents

The “inventory for the assessment of quality of life in children and adolescents” (“Inventar zur Erfassung der Lebensqualitaet bei Kindern und Jugendlichen (ILK)” [86] is a rating scale. The form is filled in by the children as well as their parents and consists of seven questions concerning the child’s mental and physical wellbeing in different areas of life. We performed the ILK before learning Chinese (pretest) and after having learned Chinese (posttest) on children and parents separately. For the children, we referred to the norm values of a representative school sample, for the parents, we referred to norm tables of representative samples based on phone interviews, as advised in the ILK manual. The child was asked the ILK-questions by an examiner and answered by pointing at one of five faces from very happy (good) to very sad (bad). The parent, on the other hand, had to choose an answer by checking a box. Similar to the child’s questionnaire, there were five possible answers to each question ranging from “very good” to “very bad.”

Two scores can be analyzed for each questionnaire: The “problem score” (PR_0-7_) consists of binary values. Possible indications for a problem are the answers ‘partly,’ ‘bad’ and ‘very bad’, which are set at 1 if one of these answers is given. When choosing ‘very good’ or ‘good,’ the dichotomous value equals 0. The PR_0-7_ is the sum of the seven dichotomous values and includes values from 0 (no area of life is considered conspicuous) to 7 (every area of life is considered conspicuous). The ILK manual suggests an overall interpretation into inconspicuous for percentile ranks (PR) ≥ 75 (sum score 0–2 or 3) and conspicuous for PR < 75 (sum score 3 or 4–7).

The QoL score 0–28 (LQ_0-28_) is a score that concerns the QoL of children and parents. Possible scores range from 0 (lowest) to 28 (highest). It is based on the same questions as PR_0-7_ and is further classified into three categories: Below average (PR ≤ 75), average (75 < PR < 85), and above average (PR ≥ 85).

#### Specific questions after the Chinese lessons

We asked the children the following questions after they had attended the Chinese lessons:

Question 1: Was it fun for you to learn Chinese? (Possible answers: yes, sometimes, no)
Question 2: Was it difficult to recall the meaning of the Chinese character? (Possible answers: yes, sometimes, no)
Question 3: Was it difficult to recall the Chinese word for it? (Possible answers: yes, sometimes, no)

### Statistical methods

All statistical analyses were performed by SPSS software (IBM Corp. Released 2021. IBM SPSS Statistics for Windows, Version 28.0. Armonk, NY: IBM Corp) licensed by the University Hospital Tuebingen. Unless otherwise stated, the Shapiro-Wilk normality test and graphical Q-Q plots were used to determine the distributions. Reading speed data were normally distributed. Therefore, we applied parametric methods for this analysis. As most of the other data were not normally distributed, we applied non-parametric confirmatory analyses consistently in all tests by the Mann-Whitney U test for independent samples. For within-group comparisons, we used the Wilcoxon signed-rank test. We report descriptive data as medians and interquartile range (IQR). Boxplots for presenting the results of each single stimulus independently (pictures, alphabetic words, and Chinese characters) were presented by pairwise-exclusion (see https://libguides.library.kent.edu/SPSS/Explore). For the sets of pictures and alphabetic word stimuli, the median result for each child and variable was calculated. This also allowed presenting one result as a boxplot for all stimuli of a set together. In the task of naming Chinese characters in German, all children gave correct answers in at least 7 out of the 12 characters. Because of more frequent mistakes in the task of naming Chinese characters in Chinese, the median result of each variable was calculated only for children with correct answers for at least 3 characters for the following reason: If a child named a character not at all, it was not possible to determine the articulation latency, the number of fixations, and the fixation duration for this stimulus. Only one child gave less than 3 correct answers and was therefore not included in this analysis.

The level of significance α was set to 0.05 (two-sided) in all statistical tests. We applied a Holm-Bonferroni adjustment to the p-values in the multiple comparison tests to avoid accumulation of Type I errors [87].

Correlation analysis was performed using the Pearson correlation if the data were normally distributed, and the Spearman correlation if this was not the case.

Regarding the ILK questionnaire PR_0-7_ (2 categories: conspicuous, inconspicuous) for the statistical difference between two test points in paired samples, we used McNemar’s test. For group comparisons we applied the Mann-Whitney U test.

Regarding the ILK questionnaire LQ_0-28_, (3 categories: below average, average, above average) a Chi-square test was performed to analyze the differences between time points. If n was < 20, we used Fisher`s exact test. For group comparisons we applied the Mann-Whitney U test.

We calculated the a priori required sample size using the software application “G*Power”, with alpha = 0.05, power = 95% and effect size Cohen’s d = 2.56 (Cohen’s d, Cohen 1992 using G*Power’s effect size calculation feature). In our previous study [30], we found a highly significant difference between children with and without dyslexia while reading text, which is why we used it as the basis for our calculations: The required sample size was computed a priori using a two-tailed model with an underlying assumed min ARE distribution. The min ARE distribution in the G*Power settings is the most pessimistic one, which then equates to an upper boundary of the calculated sample size. The total sample size is calculated as a function of power: The result for an alphabetic text reading task at a power level of 95% is a total sample size of 12 (6 in each group). Reducing the power to 80% would result in a total sample size of only 8.

## Results

Data at baseline are shown in Table 1 (see above, participants). All children had normal far and near visual acuity and normal ophthalmological findings, as required by the inclusion criteria.

### Crowding

The difference of near visual acuity between single optotypes and grouped optotypes was 1.0 log step (IQR 0.0–1.5) for group C and 1.5 log steps (IQR 1.2–1.9) for group DD. The difference between the groups of 0.5 log steps was statistically significant (U = 274.5, p = .012, Mann-Whitney U test). This group difference means half a line on the Oculus near vision chart, which cannot be considered clinically relevant in the cohort examined here. Comparing individuals, it was remarkable that we found a difference of > = 2 lines in only 1 child of group C, but in 5 children of group DD. However, we did not find any individual features in the children who showed a difference of > = 2 lines.

### Reading speed

Reading speeds assessed by the ZLT in groups DD and C were normally distributed. Reading speed for group DD was statistically significantly slower (mean 62.7 wpm, SD 25.7) than for group C (mean 125.7 wpm, SD 20.9, t (38) = 8.56, p < .001, Cohen’s d = 2.72).

### Eye tracking results

The main results of the eye movement parameters will be summarized before the detailed analysis will be reported: Statistically significant differences between the groups were found in the alphabetic word reading task for articulation latency and number of fixations. No statistically significant differences were found for CN-picture naming, and for naming a Chinese character, neither in German nor in Chinese.

All statistical values (median, interquartile range, test procedures, p-values) are shown in Table 2.

**Table 2:** Statistical data. Eye tracking.

	articulation latency (IQR) [s]		number of fixations (IQR)		fixation duration (IQR) [s]	
	DD	C	DD	C	DD	C
**alphabetic words** (DD: n = 18; C: n = 22)	1.13 (1.00–1.52)	0.84 (0.69–0.99)	3.50 (3.00–4.00)	2.00 (2.00–2.00)	0.35 (0.32–0.42)	0.33 (0.30–0.40)
MWU-test (Holm-Bonferroni correction)	U = 368, **p < .001**		U = 372, **p < .001**		U = 231, p = 1.00	
**pictures** (DD: n = 18; C: n = 22)	0.99 (0.82–1.00)	0.87 (0.79–0.99)	2.25 (2.00–3.00)	2.00 (2.00–2.00)	0.39 (0.33–0.43)	0.39 (0.35–0.44)
MWU-test (Holm-Bonferroni correction)	U = 249.5, p = 1.00		U = 264, p = .75		U = 171, p = .476	
**Chinese characters, German naming** (DD: n = 15; C: n = 21)	1.49 (1.14–1.99)	1.25 (1.08–1.68)	3.00 (2.00–3.00)	2.00 (2.00–3.50)	0.51 (0.46–0.64)	0.52 (0.48–0.74)
MWU-test (Holm-Bonferroni correction)	U = 198.5, p = 1.00		U = 183.5, p = .818		U = 118.5, p = 1.00	
**Chinese characters, Chinese naming** (DD: n = 14; C: n = 21)	1.99 (1.87–2.95)	1.96 (1.48–2.06)	3.25 (2.00–5.25)	2.0 (2.00–4.00)	0.49 (0.44–0.60)	0.73 (0.49–0.94)
MWU-test (Holm-Bonferroni correction)	U = 184, p = 1.00		U = 174.5, p = 1.00		U = 101, p = 1.00	

Reading alphabetic words, naming pictures and Chinese characters. DD: developmental dyslexia (group DD), C: control (group C), n: number of children, p = probability value, statistically significant differences are written in bold. IQR = interquartile range; s = seconds; MWU = independent samples Mann-Whitney U test with Bonferroni-Holm correction, U: Mann-Whitney test statistic; alphabetic words: Group DD with longer articulation latency (p < .001), more fixations (p < .001). Picture naming without statistical difference in all outcome variables. Naming Chinese characters in German and Chinese: No statistically significant difference between the groups in any variable.

**Fig 3:**
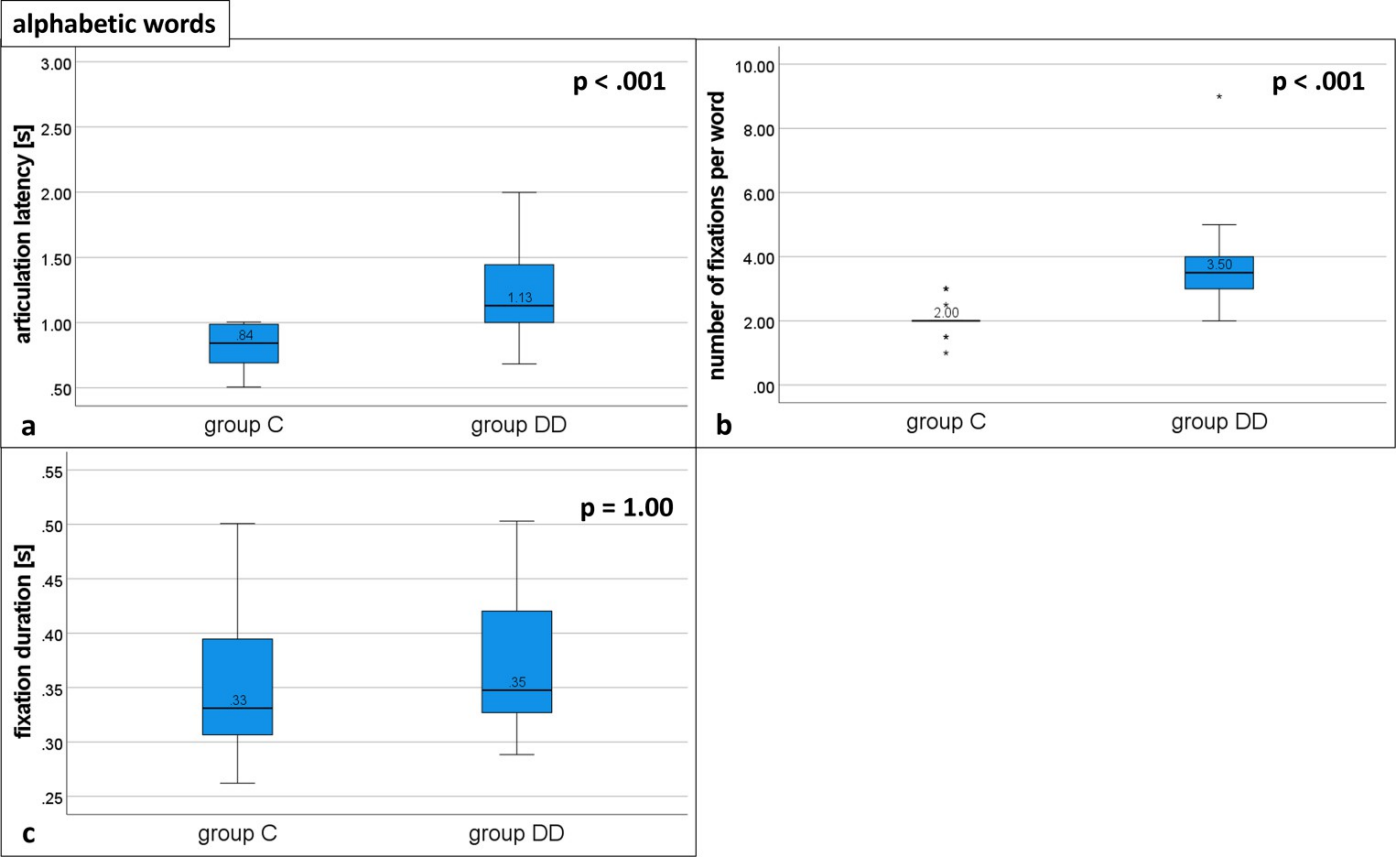
Eye movement variables during reading alphabetic words. Distribution of group DD and group C. Medians of a) articulation latency, b) number of fixations, and c) fixation duration; p = probability value. Statistically significant differences for a) and b). For details see Table 2.

**Reading alphabetic words (Fig 3 and Table 2).** The articulation latency was significantly longer in group DD (1.13, IQR 1.00–1.52) compared with group C (0.84, IQR 0.69–0.99 U = 368, p < .001; see Fig 3A and Table 2). The number of fixations per word was significantly higher for group DD (3.50, IQR 3.00–4.00) than for group C (2.00, IQR 2.00–2.00; U = 372, p < .001, see Fig 3B and Table 2).

The fixation durations did not differ between the groups (group DD: 0.35, IQR 0.32–0.42; group C: 0.33, IQR 0.30–0.40; U = 231, p = 1.00; see Fig 3C and Table 2).

#### Naming pictures (Fig 4 and Table 2)

**Fig 4:**
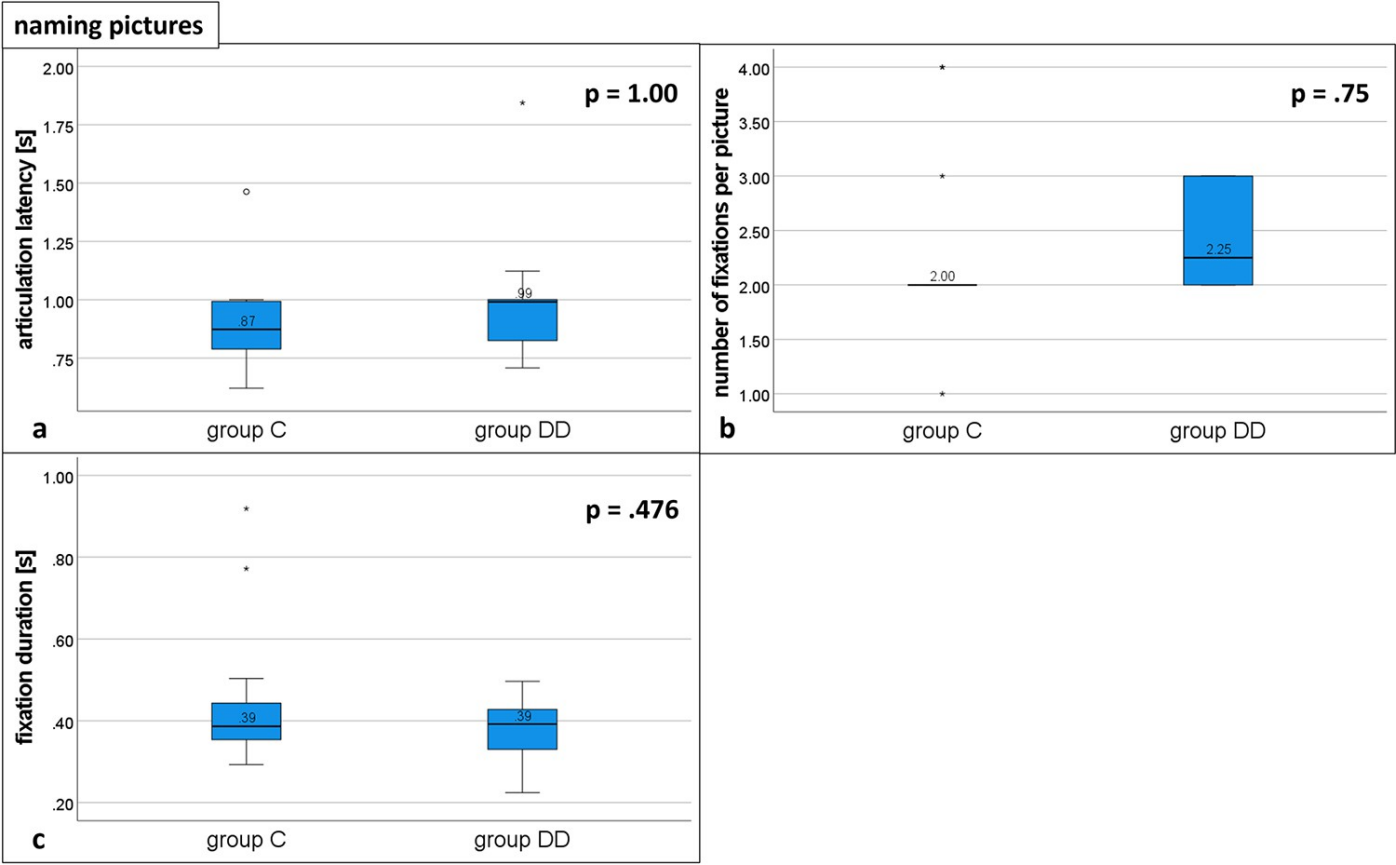
Eye movement variables during naming pictures. Distribution of group DD and group C: Medians for a) articulation latency, b) number of fixations, and c) fixation duration; p = probability value. There was no statistically significant difference between the groups for all 3 eye movement variables. See Table 2 for details.

The articulation latencies (Fig 4A) were not significantly different between the groups (DD: 0.99, IQR 0.82–1.00; group C: 0.87, IQR 0.79–0.99; U = 249.5, p = 1.00; see Table 2).

The number of fixations per picture was not significantly different between the groups, (DD: 2.25, IQR 2.00–3.00; controls: 2.00, IQR 2.00–2.00, U = 264, p = .75; Fig 4B and Table 2). Fixation durations were not significantly different between the groups (group DD:0.39, IQR 0.33–0.43; group C: 0.39, IQR 0.35–0.44; U = 171, p = .476; see Fig 4C and Table 2).

#### Naming Chinese characters in German

**Fig 5:**
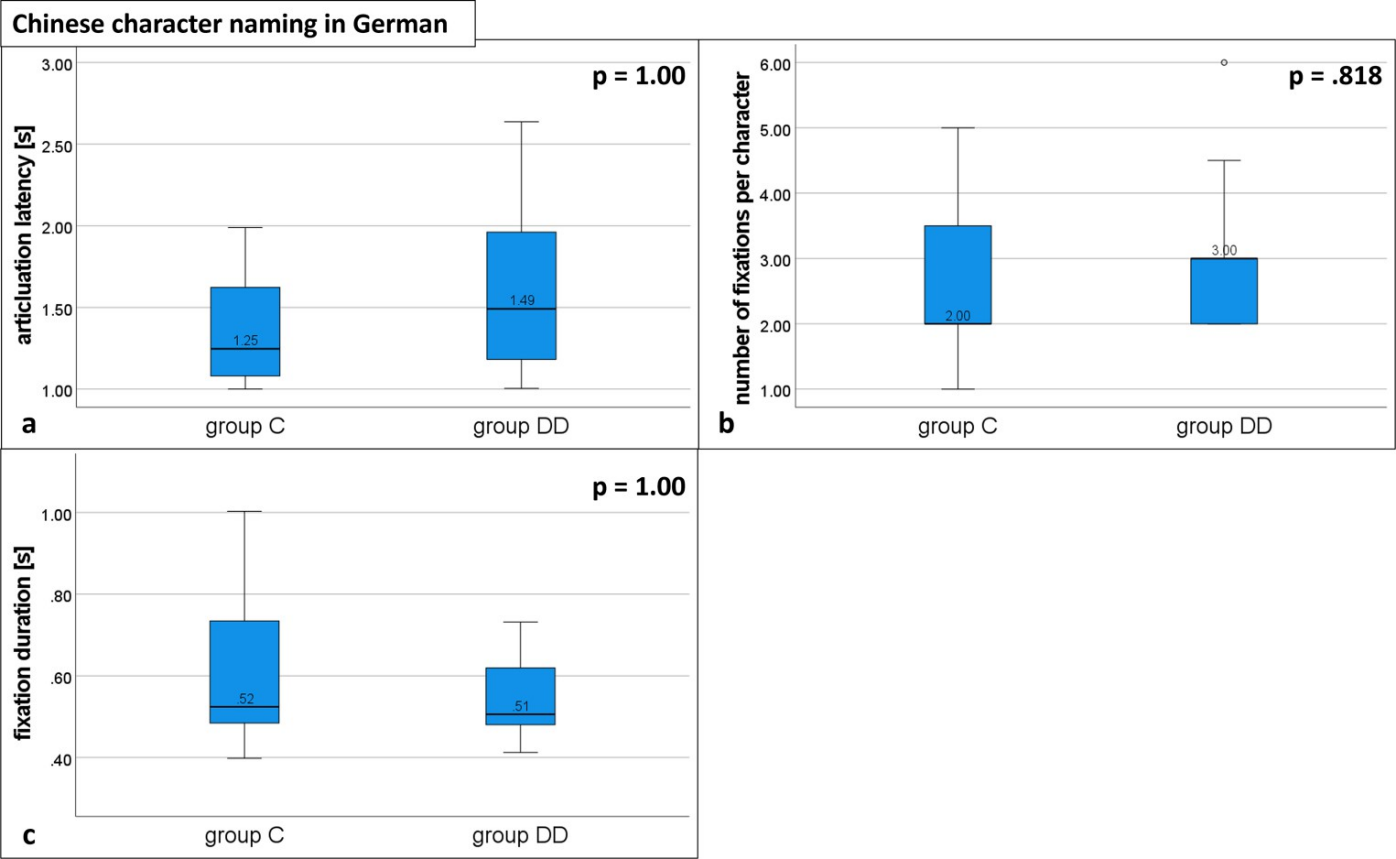
Eye movement variables during naming Chinese characters in German. Distribution of group DD and group C. Medians for a) articulation latency, b) number of fixations, and c) fixation duration; p = probability value. No statistically significant differences. See Table 2 for details.

There was no statistically significant difference between the groups for the median of all variables: Articulation latency (group DD: 1.49, IQR 1.14–1.99; group C:1.25, IQR 1.08–1.68; U = 198.5, p = 1.00; Fig 5A and Table 2), number of fixations (group DD: 3.00, IQR 2.00–3.00; group C:2.00, IQR 2.00–3.50; U = 183.5, p = 0.485818; Fig 5B and Table 2), and fixation duration (group DD: 0.51, IQR 0.46–0.64; group C:0.52, IQR 0.48–0.74; U = 118.5, p = 1.00; Fig 5C and Table 2).

#### Naming Chinese characters in Chinese

The medians of all three variables were not statistically significantly different between the groups.

**Fig 6:**
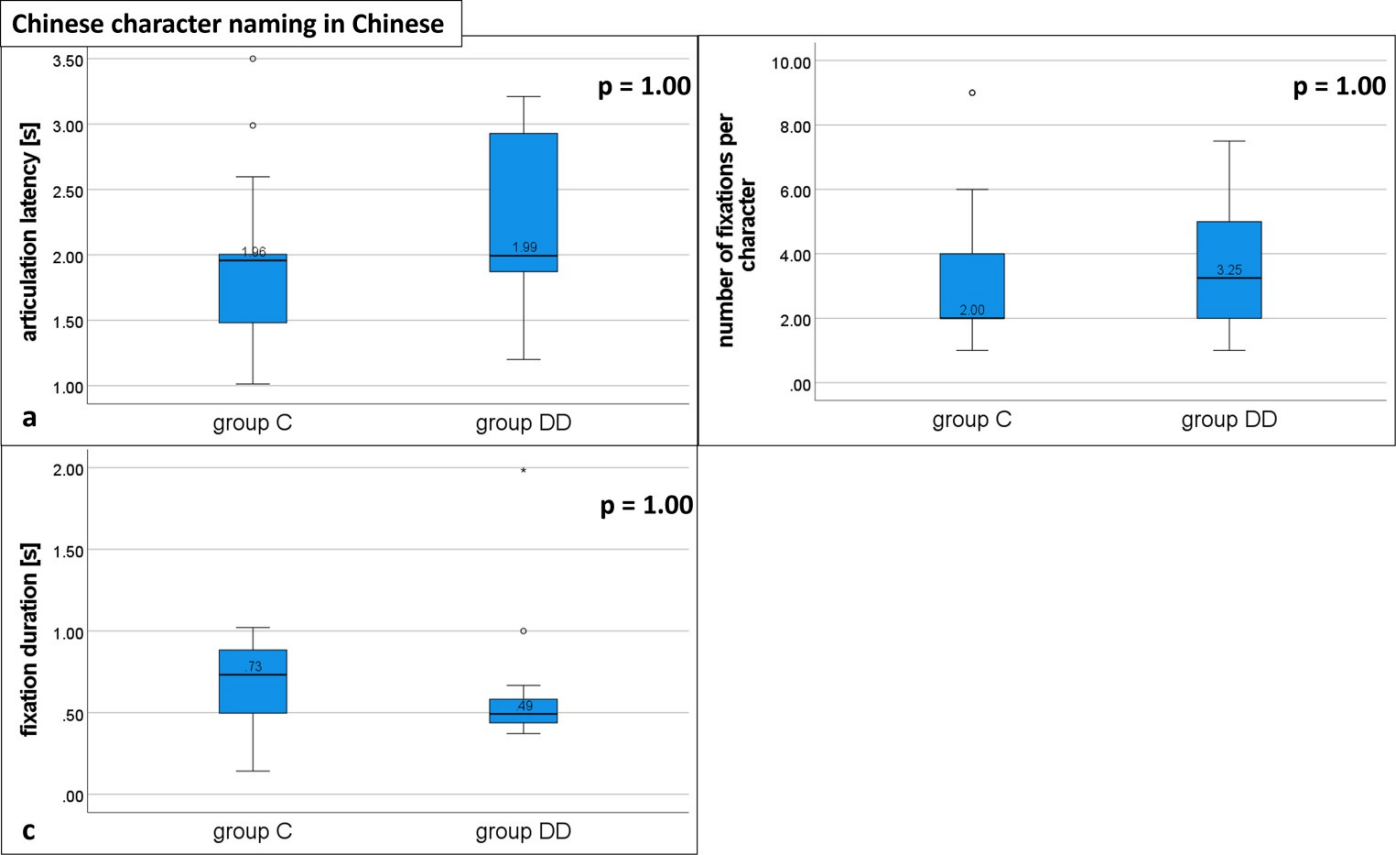
Eye movement variables during naming Chinese characters in Chinese: Distribution of group DD and group C. Medians for a) articulation latency, b) number of fixations, and c) fixation duration; p = probability value. No statistically significant differences for all three variables. See Table 2 for details.

Articulation latency did not differ between the groups (group DD: 1.99, IQR 1.87–2.95; group C: 1.96, IQR 1.48–2.06; U = 184, p = 1.00; see Fig 6A and Table 2). There was no statistically significant difference between the groups for the number of fixations (group DD: 3.25, IQR 2.00–5.25; group C: 2.0, IQR 2.00–4.00; U = 174.5, p = 1.00; Fig 6B and Table 2). The fixation duration per Chinese character was not significantly different between the groups (group DD: 0.49, IQR 0.44–0.60; group C: 0.73, IQR 0.49–0.94; U = 101, p = 1.00; Fig 6C and Table 2). Interestingly, the fixation durations were slightly longer in group C than in group DD. Even though this difference did not reach statistical significance, it hints at a strategy, which we noticed by listening to the children: When they were not sure, children in group C often made an effort to find the name, while children in group DD more often gave up earlier and said “I don`t know” (see error rate below), but in all other cases they named the character spontaneously and quickly.

As the medians for an individual child could be calculated only if at least 3 answers were correct, the incorrectly or not named characters were not included in the medians of the eye tracking analysis.

#### Correlations between the different tasks

**Fig 7:**
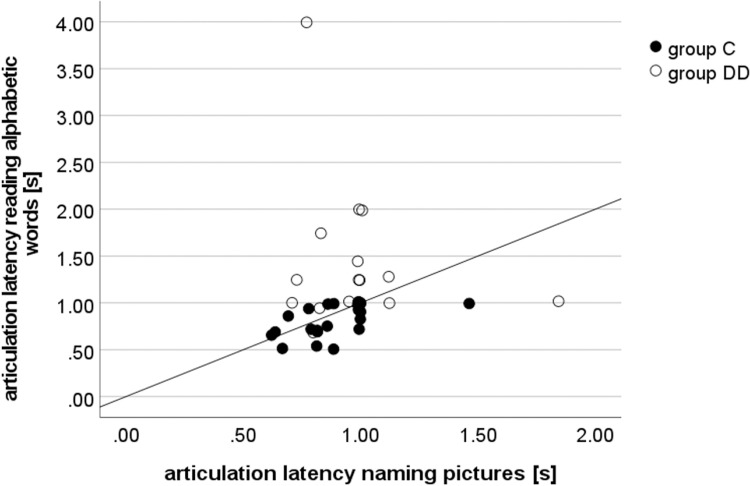
Correlation between articulation latency for pictures (abscissa) vs. alphabetic words (ordinate). For pictures, the articulation latencies are widely overlapping between the groups. For alphabetic words, the groups are to a great extend separated. Group DD is slower in word reading. Shorter articulation latencies for words than for pictures are found mainly in group C. Positive statistically significant correlation for group C: Spearman’s Rho, *r*_*s*_(20) = .619**, p = .002; no statistically significant correlation for the children with dyslexia: Spearman’s Rho, *r*_*s*_(16) = .051, p = .842.

Positive correlations with statistical significance were found for the following variables:

*Pictures vs. alphabetic words (Fig 7).* While group C showed a strong positive correlation between the articulation latencies of alphabetic words versus pictures (Spearman’s Rho, *r*_*s*_(20) = .619**, p = .002), no statistically significant correlation was found in group DD (Spearman’s Rho, *r*_*s*_(16) = .051, p = .842). The distributions of articulation latencies for pictures widely overlapped between the groups, whereas they were to a great extend separated for alphabetic words. The children with dyslexia were much slower in reading words (see also Fig 7). Only group C had shorter articulation latencies for words than for pictures.

*Chinese characters named in German vs*. *in Chinese*. There was a statistically significant correlation between articulation latency for naming in German and in Chinese for group C (Spearman’s Rho, r_s_(19) = .594**, p = .005), and group DD (Spearman’s Rho, r_s_(12) = .670**, p = .009).

The number of fixations during picture naming showed a positive and statistically significant correlation with the number of fixations during naming Chinese characters in Chinese for group DD (Spearman’s Rho r_s_ = .540*, p = .046), but not for group C (Spearman’s Rho r_s_ = .390, p = .081).

### Error rates

During naming alphabetic words and pictures, the children of both groups did not make any mistakes. During naming Chinese characters group DD made significantly more errors than group C, which was much more pronounced in Chinese naming.

The **percentage of correct answers** during naming the Chinese characters in **German** was very high in both groups, but significantly lower in Group DD (86.67, IQR 66.67–100) than in group C (95.24, IQR 91.67–100; Wilcoxon p = .015). This difference increased during naming the characters in **Chinese** (group DD: 56.67, IQR 41.67–73.06; group C: 83.77, IQR 74.78–95.24; Wilcoxon p = .003). It is evident that the children of **both** groups made more errors, when they had to name the Chinese characters in Chinese than in German. The children of group DD failed to name the Chinese character more often, especially in Chinese. However, **if** they named the character, they performed as well as group C, as shown in the three eye movement variables above, which represent only the correct answers. Interestingly, group ***DD*** answered”I don’t know” more often during Chinese naming (23.89%) than answering incorrectly (15.0%), but much more frequently than the control group (7.14%)—see Table 3.

**Table 3:** Error rate.

*Chinese characters naming tasks*	*percentage of number of children who answered correctly*, *false*, *or "I don’t know" Median percent (IQR)*					
	*group C*			*group DD*		
	*correct*	*false*	*I don´t know*	*correct*	*false*	*I don´t know*
*German naming*	***95*.*24 (91*.*67–100*.*00)***	*0*.*00 (0*.*00–4*.*76)*	*2*.*38 (0*.*00–4*.*76)*	***86*.*67 (66*.*67–100*.*00)***	*6*.*67 (0*.*00–13*.*33)*	*3*.*33 (0*.*00–18*.*33)*
*Chinese naming*	***83*,*77 (74*.*78–95*.*24)***	*4*.*76 (0*.*00–9*.*42)*	*7*.*14 (0*.*00–17*.*21)*	***56*.*67 (41*.*67–73*.*01)***	*15*.*00 (6*.*67–25*.*00)*	*23*.*89 (7*.*78–45*.*00)*

The percentage of children who answered correctly, false or answered “I don’t know” during the Chinese character naming task in German and in Chinese. Group DD made statistically significant more errors in both tasks, which was more pronounced in the Chinese naming task.

### Quality of life questionnaires

#### Problem score PR_0-7_

There were no statistically significant differences in the problem score PR_0-7_ between pre- and posttests, neither between the children`s and parents`reports, nor between the groups. From the children`s perspective, they were inconspicuous in 88.9% of group DD and in 95.5% of group C at pretest. For details see Table 4.

**Table 4:** Statistical data of problem score PR_0-7_.

		PR_0-7_ pretest		PR_0-7_ posttest		pre-post comparison McNemar-Test	children-parents comparison (pretest) McNemar-Test	children-parents comparison(posttest) McNemar-Test	group DD/ group C comparison (pretest) Mann-Whittney U	group DD/ group C comparison (posttest) Mann-Whittney U
		conspicious n (%)	inconspicious n (%)	conspicious n (%)	inconspicious n (%)					
Group DD	children (n = 18)	2 (11.1%)	16 (88.9%)	2 (11.1%)	16 (88.9%)	p = 1.00	p = .219	p = .219	U = 185, p = .579	U = 176, p = .196
	parents (n = 18)	6 (33.3%)	12 (66.7%)	6 (33.3%)	12 (66.7%)	p = 1.00			U = 168, p = .464	U = 150, p = .110
Group C	children (n = 22)	1 (4.5%)	21 (95.5%)	0 (0.0%)	22 (100.0%)	p = 1.00	p = .250	p = .50		
	parents (n = 22)	4 (18.2%)	18 (81.8%)	2 (9.1%)	20 (90.9%)	p = .625				

The 2 categories of answers are compared between the groups (DD = dyslexia, C = control), between children (blue boxes) and parents (white boxes) and between pre- and posttest. n: number of subjects; p = probability value; U: Mann-Whitney U test statistics. No statistically significant differences between pre- and posttests, neither between the children`s and parents`reports, nor between the groups. From the children`s perspective, they were inconspicuous in 88.9% of group DD and in 95.5% of group C at pretest.

#### Quality of life score LQ_0-28_

The difference between time points (pre/post) was not statistically significant for children and parents of group DD. In group C, it was not significant for children, but for parents, who revised their judgement to a better category at posttest. For details see Table 5.

**Table 5:** Statistical data of quality of life: LQ_0-28_.

		LQ_0-28_ pretest			LQ_0-28_ posttest			pre-post comparison	children/parents comparison (pretest)	children/parents comparison (posttest)	group DD/ group C comparison (pretest) Mann-Whittney U—Test	group DD/ group C comparison (posttest) Mann-Whittney U—Test
		below average n (%)	average n (%)	above average n (%)	below average n (%)	average n (%)	above average n (%)					
Group DD	children (n = 18)	1 (5.6%)	15 (83.3%)	2 (11.1%)	0 (0.0%)	17 (94.4%)	1 (5.6%)	χ^2^(2, n = 18) = 8.471, p = .167 (Fisher’s exact)	χ^2^(2, n = 18) = 3.378, p = .331 (Fisher’s exact)	χ^2^(1, n = 18) = 4.07, p = 1.00 (Fisher’s exact)	U = 191.5, p = .949	U = 119, **p = .011**
	parents (n = 18)	5 (27.8%)	13 (72.2%)	0 (0.0%)	5 (27.8%)	13 (72.2%)	0 (0.0%)	χ^2^(1, n = 18) = 0.516, p = .583 (Fisher’s exact)			U = 133.5, **p = .048**	U = 113, **p = .007**
Group C	children (n = 22)	1 (4.5%)	18 (81.8%)	3 (13.6%)	0 (0.0%)	12 (54.5%)	10 (45.5%)	χ^2^(2, n = 22) = 2.057, p = .368 (Chi-Square)	χ^2^(4, n = 22) = 11.917, **p = .010** (Chi-Square)	χ^2^(2, n = 22) = 0.892, p = 1.00 (Chi-Square)		
	parents (n = 22)	4 (18.2%)	11 (50.0%)	7 (31.8%)	1 (4.5%)	15 (68.2%)	6 (27.3%)	χ^2^(4, n = 22) = 18.267, **p = .001** (Chi-Square)				

The 3 categories of answers are compared between the groups (DD = developmental dyslexia, C = control), between children (blue boxes) and parents (white boxes) and between pre- and posttest.

n: number of subjects; p = probability value; U: Mann-Whitney U test statistics. χ^2^: Pearson Chi-Square test statistics.

No significant difference between pre- and posttest for children and parents of group DD. In group C, no significant difference for children, but for parents, who revised their judgement to a better category at posttest. Comparison between children and parents: statistically significant difference only for parents of group C at pretest. At posttest, no statistically significant differences between children`s and parents`judgements. No statistically significant difference between the groups at pretest for the children, but only for the parents, due to a higher ranking in group C. The significant difference between the groups at posttest was based mainly on the generally higher ranking in group C (children and parents).

The comparison between children and parents showed that the children always perceived their QoL better than their parents, but this difference did not reach statistical significance, except for group C at pretest. This latter statistically significant difference was mainly caused by the parents, who rated their children more often as “below average” and less often “at average”. At posttest, there were no statistically significant differences between children`s and parents`judgements.

The difference between the groups at pretest was not statistically significant for the children, but only for the parents, due to a higher ranking in group C. The significant difference between the groups at posttest (children and parents) was based mainly on the generally higher ranking in group C, which improved from “average” to “above average” in the children and from “below average” to “average” in the parents (see Table 5).

#### Questions regarding Chinese lessons

**Fig 8:**
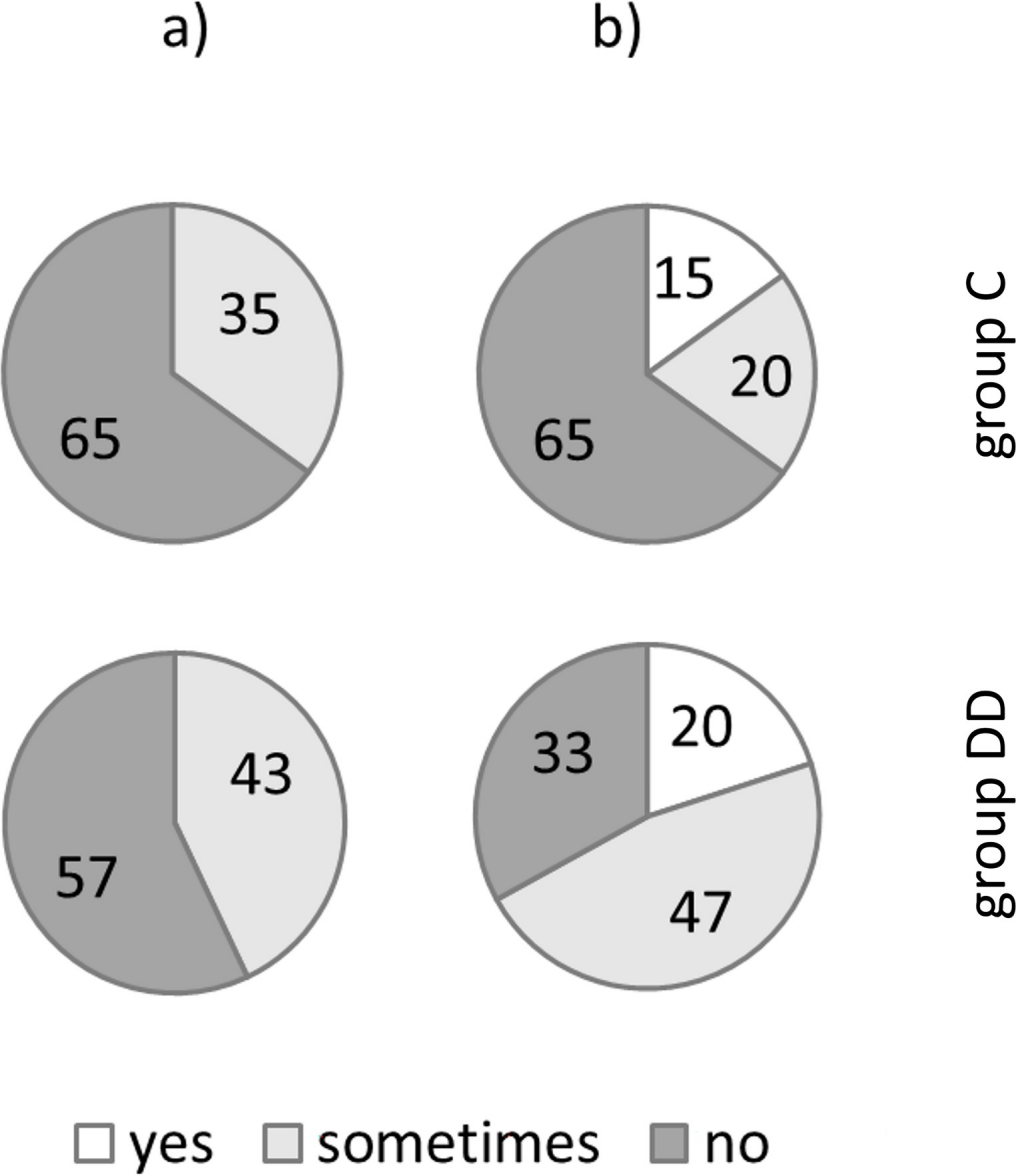
Survey regarding Chinese lessons. Distribution of perceived difficulties while learning Chinese for the children of groups C and DD. Two questions: a) Was it difficult to recall the meaning of the Chinese character (if it was to be named in German)? b) Was it difficult to find the Chinese word for the character? The answers could be: “yes” (white), “sometimes” light grey, and “no” (dark grey). The subjective reports were in agreement with the objective data: The children with dyslexia reported more difficulties, but only for naming in Chinese.

After the Chinese lessons, a survey explored the children’s subjective assessment of learning Chinese (see Fig 8).

All children of both groups reported that they had fun learning Chinese. When they were asked whether it was difficult to recall the meaning of the Chinese characters (to be named in German, Fig 8A), both groups answered similarly, as 35% in group C and 42.9% in group DD reported that they sometimes perceived this task as difficult. On the other hand, 65% of group C and 57.1% of group DD did not report difficulties recalling the German meaning. The analysis showed no significant difference between the groups (Fisher’s exact test: p = .728). Being asked whether it was difficult to recall the Chinese word (Fig 8B), 65% of group C reported no difficulties, but only 33.3% of group DD found this to be true. Almost half of group DD (46.7%) mentioned that they sometimes had trouble recalling the Chinese word, compared with 20% of group C. The answer ‘yes’ was given by 15% in group C and 20% in group DD. However, the difference was not statistically significant (Chi-square test: p = .154)

In summary, the perceived difficulties are partly in agreement with our objective findings. Looking at Chinese characters that were to be named in German, there was no significant difference between the two groups. Regarding the task of naming Chinese characters, the analysis showed similar results with no statistically significant difference between the groups. However, combining the answers “yes” and “sometimes” indicates that more children with dyslexia had to struggle while naming characters in Chinese (Fisher’s exact test: p = .092).

## Discussion

These are the main results of the present study:

1. Marked group differences in alphabetic word reading: Prolonged articulation latencies and higher number of fixations in group DD as compared to group C.
2. No group differences for naming pictures and Chinese characters in German and in Chinese in the eye movement variables (only correct answers included).
3. High percentage of correct answers in both groups for **German naming** of the Chinese characters, lower percentage in group DD.
4. Increased error rate in both groups for **Chinese naming** of the Chinese characters, more pronounced in group DD.
5. QoL—From the children`s perspective: no difference between the groups at pretest, but at posttest. From the parents`perspective: Lower QoL of their children perceived by parents of group DD, compared with parents of group C at pre- and posttest.

### Patient selection

We did not consider possible subtypes of dyslexia in our study, because the phonological type of dyslexia is predominant in regular orthographies such as German. This means that most German words can be decoded by regular grapheme-phoneme correspondence [10, 23, 88, 89]. In contrast, English contains many irregularly spelled words that require lexical-orthographic processing, which has been found to be impaired in the surface subtype of dyslexia [90]. Based on studies in an indigenous language of India (Kannada), Karanth [91] reported that “in languages that not only lack irregular words, but in which alternative spellings of a sound or word are not possible, surface dyslexia cannot be observed”. All single alphabetic words used in the current study were of regular orthography and did not require lexical-orthographic processing [45, 90, 92]. The primary phonological deficit of the children shown here and in our previous studies is indicated by an increased struggle with words of higher phonological difficulty [72, 73].

### Potential crowding

We did not find a **visual deficit** in the children of both groups, which might have influenced the discrimination of the Chinese characters. To assess a **potential crowding** effect, we examined the difference of near visual acuity between single optotypes and grouped optotypes. The **group difference** of 0.5 log steps means only half a line on the Oculus near vision chart. Even though this difference was statistically significant, it cannot be considered clinically relevant in the cohort examined here. Looking at **individuals**, it was remarkable that we found a difference of = > 2 lines in only 1 child of group C, but in 5 children of group DD. This could be a hint at increased occurrence of crowding in children with dyslexia. However, we did not find any individual features in the children who showed a difference of > = 2 lines. Enhanced crowding has been reported in previous studies [31–36]. But these findings are still controversial and have been questioned in recent publications [20, 36, 42], who have hypothesized that a visual deficit may only be present in subgroups with DD.

### Alphabetic words

Our results revealed that reading performance of alphabetic words was significantly impaired in children with dyslexia. Specifically, we observed a lengthening of the articulation latency and an increase in the number of fixations. In contrast, the duration of the fixation period did not differ between the groups. This indicates that children with dyslexia process words in smaller units, which has also been interpreted as a strong reliance on sub-lexical reading [93].

Our results agree with the phonological deficit hypothesis in alphabetic languages, which indicates that the difficulty in converting graphemes into phonemes is especially found in languages with regular orthography, such as German [5–8]. Furthermore, a deficit in the phonological loop of working memory can also be of influence, if simultaneous storing and processing of phonological information is required [14].

The increase in articulation latency and number of fixations has been reported in the literature [75, 93, 94] and is consistent with our previous studies [73, 74]. In contrast to the normal fixation durations in the present study, these were prolonged in our earlier studies [72–74], which was also reported by other authors [75, 93, 95]. We assume that this discrepancy might be attributed to the different level of difficulty of the reading material.

The influence of typeface on reading performance in alphabetic languages is reported controversially in the literature. Some authors claim a superiority of a sans serif font (Arial) for reading on a screen [96], while others did not find a significant difference between a serif and a sans serif font in children with dyslexia [97], as well as in an early study with 900 not reading-impaired people [98], and in studies on patients with macular degeneration, where not reading-impaired persons served as controls [61, 99]. Nedeljković and co-workers [100] found that the familiarity with a font influences reading performance more than other factors. We do not think that the use of a serif typeface (Times New Roman) for the alphabetic words caused a bias in our study, because both groups (with and without dyslexia) were examined with the same font. Furthermore, we do not think that the serif font had an essential effect on reading performance for alphabetic reading, because the eye movement pattern of alphabetic words was extremely impaired in the children with dyslexia compared with those without. In addition, we chose this serif font deliberately in order to provide the same kind of display for eye tracking with both methods, and because we have used the serif font in all our previous studies involving the SLO.

### Picture naming

We did not use a rapid automatized naming (RAN) task, because RAN requires fast automatized access to the phonological representation of serially (repeatedly) displayed visual stimuli. A RAN deficit has been reported in many studies, but its interpretation is still controversial [16–23]. We wanted to examine the performance in a naming task that does not require automatization and grapheme-phoneme conversion, where the primary process is visuo-spatial, but not letter-mediated. This is why we applied a confrontational naming (CN) task, which requires naming pictures without time pressure and without serial presentation. We found in a previous study that children with DD performed naming pictures as well as their age-matched controls [30]. In the present study, we aimed to verify this result and to examine whether unimpaired picture naming might be used as a predictor for good performance in a non-alphabetic, logographic script such as Chinese. In the CN-task used here, the eye movement variables did not differ between the groups. The lack of a correlation between CN and alphabetic word reading in group DD (Fig 7) indicates that the two tasks require different skills and shows the problem with letter-mediated stimuli that require phonological decoding.

In contrast to our current and previous findings that were based on eye movement recordings, a study about children with dyslexia in English reported a CN deficit–assessed as percentage of correct responses, but used a different study design and a larger number of stimuli [44]. However, the same study [44] showed a remarkable overlap between the children with dyslexia and two other groups (garden variety poor readers and not reading-impaired children). In addition, a CN-deficit was observed only for pictures associated with long, low frequency words. This was interpreted as a deficit in retrieving the phonological code of the word belonging to the picture [44]. This discrepancy may be influenced by the fact that English speaking children are generally not used to long words, whereas German contains many long words, a fact to which German children are accustomed. Ziegler et al. [45] described a pretest that used a naming task without time pressure in order to assess familiarity and correct naming of the pictures. They did not find any deficit in this pretest, which is in agreement with our results. We assume that confrontational picture naming is processed via the visuo-spatial sketchpad of working memory with direct access to the semantic system. Our results indicate that the visuo-spatial sketchpad is unimpaired in children with dyslexia in an alphabetic language, which has also been mentioned by other authors [14, 29].

The positive statistically significant correlation between the number of fixations during naming pictures and Chinese characters in Chinese observed in group DD might be an indication of a predictive value for good performance in a non-alphabetic, logographic script such as Chinese. However, this slight correlation cannot yet be evaluated due to the low number of pictures used in the current study and should be examined in future studies.

Furthermore, the normal CN-picture naming performance contradicts the assumption of an underlying oculomotor dysfunction in children with dyslexia, which we also found in previous studies [101, 102].

### Naming Chinese characters

Since we were aware of the phonological deficit of our children with dyslexia, we wanted to avoid a primary disadvantage for the children by requiring phonological processing. This is why we explained the required task just verbally. Thus, we did not use any letter-mediated information during the lessons, neither alphabetic words to explain the meaning, nor the alphabetic transcription of the Chinese characters (Hanyu Pinyin) to explain the Chinese pronunciation. In addition, we let the children use a learning app, which also did not contain any letters, but showed a picture corresponding to the Chinese character that showed the meaning and played audio for the Chinese pronunciation. We surmised that this approach might make learning a logographic script, such as Chinese, easier for the children.

For native speakers, reading Chinese characters is an interplay between phonological and semantic processing [103]. The mapping between spelling and reading of Chinese characters is rather systematic. A high percentage of Chinese characters consist of both a phonetic radical **and** a semantic radical (e.g. 妈 mā, "mother": semantic radical "female" + phonetic radical "mǎ"). Due to the restrictions of the children with DD, we intended to avoid any phonological reference during the instruction. Neither was the alphabetic transcription of the Chinese characters (Hanyu Pinyin) used, nor were the phonological aspects of the Chinese characters introduced. [Only two of the stimuli contained (different) phonological radicals at all.] In order to strengthen and enhance the visual capacities of the children, the pictorial content of the Chinese characters was brought into focus. The majority of the chosen stimuli were basic characters that cannot be divided into sub-graphemes, and they originated from pictograms. Those characters containing semantic radicals were analyzed and memorized with respect to structure and semantics. Taken these considerations into account, neither the mispronunciation in Chinese nor the misnaming in German can be directly related to phonological processing. They might rather be related to memory capacities, cognitive or psychological factors (e.g. attention, motivation, language experience).

### Do the Chinese and alphabetic writing systems require different skills?

Looking at the eye movement variables during naming Chinese characters in German and in Chinese, there was no significant difference between the two groups regarding articulation latency, numbers of fixations and fixation duration. This suggests that children with dyslexia may process the Chinese characters in a way that is similar to the way members of the control group do it when focusing on their meaning. The similar eye movement pattern found in this study may be based on a shared logographic/visual phase at the beginning of learning the semantics of Chinese characters, as described by Yang [53] and Siok and Fletcher [48]. Morphological awareness has been reported as a major component of Chinese reading [49, 51, 55–61] and seems not to be compromised in German children with dyslexia when reading single logographic Chinese characters in German, since recognizing the meaning of Chinese characters can be unimpaired in children with dyslexia in an alphabetic language. Thus, our findings support the hypothesis that different skills are needed for alphabetic versus logographic languages, which agrees with several previous studies [46–48].

The method of learning a language, i.e. whether a word is processed visually as a whole, or as grapheme-to-phoneme conversion, involves different neural networks, which has been previously studied by introducing an artificial language [104–106]. Whereas perceiving a whole word relies more on brain regions in the ventral pathway, grapheme-to-phoneme mapping has been shown to be more dependent on dorsal pathways [104].

Studies of deficits of the visual attention span (VAS) have shown a correlation with dyslexia, although this is still a matter of debate. Recent research suggests that the VAS affects reading ability and fluency in Chinese [107–109] and has been associated with dyslexia in Chinese children [110]. It has also been reported that applying training to the VAS improved reading performance in Chinese children with dyslexia [111]. Regarding alphabetic languages, however, there is no consensus on whether the VAS is a cause or a consequence of dyslexia [39, 112, 113], or whether it is associated with dyslexia at all [36, 114, 115]. Ziegler et al. [20] discovered in their study, that the VAS deficit in children with dyslexia disappeared as soon as letter strings were replaced with symbols without a phonemic code.

Conversely, reading alphabetic languages requires mapping a sound to a letter string in an analytic manner, and a weakness in this skill is generally accepted as the main cause of dyslexia in alphabetic scripts. This is supported by our present study. In Chinese readers, visual-orthographic knowledge and especially morphological awareness are important for successful reading [49, 51, 55–61].

There are reports on children with dyslexia who show reading problems only in one writing system and not in the other: In contrast to English, poor Chinese readers exhibited poor performance in copying Chinese characters, which plays an important role for Chinese literacy skills [63]. Poor English readers and poor readers in both Chinese and English, showed deficits in phoneme awareness and in RAN tasks [63].

### Why did German children with dyslexia experience difficulties when naming Chinese characters, especially in Chinese?

If a child in group DD was able to name the Chinese character, then performance was as good as in members of group C–as shown by the eye movement patterns.

The percentage of correct answers was high in both groups for German naming (DD: 87%; C: 95%) and decreased for Chinese naming (DD: 57%; C: 83%). However, the children in group DD had a significantly higher error rate in both Chinese character naming tasks, but much more marked in Chinese naming.

When we looked at the incorrect answers, we found that both groups performed quite similarly for German naming, although the difference in error rate was statistically significant. Interestingly, during Chinese naming group DD answered”I don’t know” more often (23.89%) than answering incorrectly (15.0%), and much more frequently than the control group (7.14%)—see Table 3.

The higher rate of false and especially not-named Chinese characters could be influenced by various causes: Firstly, the high rate of not-named characters could indicate a memory deficit. Secondly, another aspect could be psychological factors, like a lower level of confidence (see paragraph “QoL” below). Thirdly, the phonological deficit might become “manifest” only in the more challenging task of Chinese naming (see below).

Behavioral research in the field of dyslexia in alphabetic languages [116–121], as well as studies on Chinese children with dyslexia learning English [50, 122, 123] points to the fact that children with dyslexia in their first language also have difficulties in learning a second language. However, this has been found in alphabetic languages, where the problem of the phonological deficit involves both alphabetic languages, albeit to a different degree, which depends on the regularity of the language [116–121].

A study on Chinese-English readers with difficulties in either Chinese or English only, or both, found that poor readers of both languages showed difficulties in phonological and morphological awareness. Children with dyslexia in English only had significantly poorer phonological awareness compared to the poor Chinese-only readers, whose average tone awareness was also lower compared to unimpaired controls [124]. The authors assume that the linguistic features of the particular writing system influence the degree of importance for metalinguistic skills for the different writing systems. Another study on children with reading problems only in one writing system and not in the other reported similar results: in contrast to English, poor Chinese readers exhibited poor performance in copying Chinese characters, which plays an important role for Chinese literacy skills [63]. Poor English readers and poor readers in both Chinese and English, showed deficits in phoneme awareness and in RAN tasks [63].

We assume that the fact that the children with dyslexia struggled more than those in group C in naming Chinese characters, especially in Chinese, can be influenced by their phonological deficit. Different aspects of the phonological deficit need to be considered to clarify its role: In learning a foreign language, working memory, and especially its subunit, the “phonological loop”, plays an important role in foreign language vocabulary language acquisition [14, 125]. The phonological loop contains the short-time storing (phonological buffer) and the active maintaining of the information (phonological rehearsal), which has to work simultaneously [13, 14, 125]. A weakness of the phonological loop would imply problems in refreshing the information and, thus, in consolidating the lexical label of the word they just learned and access to it. However, we did not test this aspect in our study. It has been reported that children with a diminished phonological memory and processing had difficulties learning a foreign language [126]. Fischbach et al. [15] showed that 8–11 years-old German-speaking children with dyslexia had the most pronounced difficulties with phonological rehearsal.

Hu et al. [127] reported that Chinese dyslexic children have deficits in both verbal short-term memory and verbal working memory. Moreover, it was found that another subunit of the central executive of working memory, the visuo-spatial sketchpad, does not play a relevant role in children with dyslexia in alphabetic languages [14, 29]. This unimpaired ability, however, might enable these children to learn Chinese characters more easily than alphabetic words. When the children in our present study were asked to name Chinese characters in German, they had the option to circumvent the phonological loop via the visual-semantic system. A possible role of the visuo-spatial sketchpad and acquisition of a novel script was discussed by Baddeley [14]. Indeed, we found in a previous study that children with and without dyslexia were able to learn the letters of a new alphabetic script (Cyrillic rather than Latin). However, reading performance in the group with dyslexia was impaired, compared with the control group, because this task also required phonological processing [128].

In contrast, a single Chinese character represents a meaning, which allows primary visual processing with direct access to the semantic system. Therefore, naming it in German was not a major problem for our children with dyslexia. When Chinese naming was required, the children with dyslexia performed worse, indicated by a significantly lower percentage of correct answers with a markedly increased difference compared with group C. One possible explanation might be their underlying phonological deficit. However, the role of phonological awareness in reading Chinese still remains unclear. On the one hand, phonological awareness seems to be less important when reading Chinese [63, 64, 124, 129]. Chinese immigrants, who were new to an English-speaking country, showed no correlation between their performance in English and phonological awareness. On the other hand, such correlation has been found in long-term Chinese immigrants [130], which indicated that they initially tried to visually read English words as a whole and only later by phonological decoding.

Kalindi et al. [63] emphasized the importance of phoneme awareness in English, but not in Chinese and the importance of copying skills in Chinese, but not in English.

Other authors have pointed out that phonological awareness can, at least in part, be a universal predictor of reading performance and, thus, is not only relevant in alphabetic languages, but also in Chinese [80, 103, 122, 131–134]. After studying a variety of writing systems, Navas et al. [8] concluded that a phonological processing deficit is likely to play a universal role in dyslexia. Perfetti et al. suggested that the use of phonology is a general characteristic of reading across different writing systems. They found early (“prelexical”) activation of phonology in English and later (“lexical”) activation in Chinese, i.e. the difference lay in the time course of activation. They concluded that visually based spelling representations may reduce the role of phonology in recognizing words, but do not entirely eliminate it.

Cheung et al. [132] found that speech perception, as well as perceived tone and aspiration, differed in Chinese children with dyslexia compared with control subjects. Goswami et al. [80] compared English, Spanish, and Chinese children with dyslexia, and discovered that the rhythmic timing and syllable perception, which are part of phonological processing, was impaired in all languages. It should be pointed out, though, that we focused on eye movement patterns in the present study. When a child was supposed to name a character in Chinese, we did not analyze whether the correctly identified meaning was given in the proper Chinese articulation.

A recent study showed an interaction between phonological and semantic processing in reading Chinese characters [103], which was interpreted as a universal connectionist model of visual word recognition across languages.

Neuroimaging studies in people without dyslexia revealed that one’s first language influences the learning of a second language [104, 135, 136]. Our study tried to eliminate this factor by deliberately avoiding a phonetic transcription of Chinese characters, like Pinyin. Moreover, there is evidence supporting an assimilation/accommodation pattern when learning Chinese. In other words, while Chinese people used similar brain activation patterns for Chinese as for English (assimilation), readers of the English language recruited further brain areas similar to those activated by Chinese readers (accommodation) [104, 137]. Nevertheless, having learned an alphabetic language that is based upon phonetics may influence the approach taken by English readers to learning Chinese.

Furthermore, neuroimaging studies on dyslexia have shown at least a partial resemblance of brain activation in English and Chinese children with dyslexia in their native language [138–140]. Along with other elements, working memory has been found to be an important factor for learning new languages [141] and has been shown to be impaired in, both, Chinese and English children with dyslexia [138, 142, 143].

Altogether, we assume that German naming of Chinese characters mainly involves semantic processing, while for Chinese naming an interaction of semantic and phonological processing is required.

### Quality of life questionnaires

The difference between the groups at pretest showed no statistically significant difference from the children`s perspective, however a slight, but statistically significantly better rating by the parents of group C, which was mainly due to a higher percentage of “above average” answers compared with those for group DD. This result supplements research that investigated perceived anxiety, depression and somatic symptoms in developmental dyslexia. Giovagnoli et al. [144] found that these signs did not differ between children with dyslexia and control subjects between the ages 8–11. The present study included children between 9 and 11 years of age, and thus agrees with the findings by Giovagnoli et al. [144]. Similarly, Balazs et al. [145] reported that children with dyslexia and controls did not differ in their self-reported QoL. During adolescence (ages 11–16), however, patients with dyslexia experienced significantly more internalizing symptoms due to increased anxiety and low levels of self-esteem and self-efficacy [144]. This can be interpreted as a sign of lower QoL, given the described (negative) correlation between depressive symptoms and QoL [86, 146]. It seems that during childhood, children with dyslexia perceive their QoL at least not predominantly in terms of dyslexia but are at a higher risk for a decrease in QoL as they get older. A certain indication for a reduced confidence of the children of group DD might be that they gave up earlier in a challenging task such as naming Chinese characters, especially in Chinese.

At posttest, the difference between the groups was statistically significant for children and parents because of the higher percentage of “above average” answers in group C.

The pre-post difference was statistically significant only from the perspective of parents`of group C, whereas the children of both groups and the parents of group DD did not show significant differences between the pretest and posttest phases, neither in PR_0-7_, nor in LQ_0-28_. The failure to perceive an increase in QoL in children with dyslexia could be due to the short duration of the Chinese lessons. It could also have been caused by the higher expectations that the parents´ of group DD had regarding their children’s ability to learn Chinese, which were not fully met. A further possible explanation is a general uneasiness with respect to learning and dealing with language. Children with dyslexia often struggle in school in general, because they have low self-esteem and feel isolated [147].

However, when the children in this study were asked about their experience during the Chinese lessons, all of them reported that they had fun learning Chinese. Based on their active participation in the lessons, we had the impression that they were highly motivated. With methods of discovery, holistic and cooperative learning, the children’s natural curiosity and desire to learn were instigated. The combination of learning characters through storytelling stimulated the visual capabilities of the children with and without dyslexia. At the closing lesson, the children proudly presented the works created during the course to their parents.

It is known that protective factors regarding self-esteem include positive relationships with peers as well as social and family support [78, 144, 148]. Fostering equal conditions such as these lessons might have a long-term positive impact on the child’s self-image and future. Parents of children with dyslexia reported a significantly lower QoL for their children compared with parents of children without dyslexia. Again, this fact might be based upon the constant struggle and different expectations between children with dyslexia and their parents. There is a general disadvantage for children with dyslexia in school and other areas of life, where there is a constant demand for reading and writing. Parents may be more aware of the possible detriments caused by a diagnosis of dyslexia. In addition, research shows that dyslexia is, at least in part, due to genetic factors [149, 150]. Consequently, parents of children with dyslexia are more likely to experience their own difficulties with reading and spelling, which in turn makes them even more susceptible to problems that their children experience. After all, teaching a language course for two weeks does not change the host of obstacles that children with dyslexia have to overcome in their everyday life.

Overall, there was no statistically significant difference between children and parents regarding PR_0-7_, although children with DD in general tended to report fewer problems or classify themselves as inconspicuous than did their parents. Matthiä [151] found similar results when they gave the ILK to children with dyslexia and their parents.

Regarding LQ_0-28_, the parents and children differed significantly, with the children showing a higher score than the parents. This discrepancy has been described before by Balazs et al. [145]. There was no significant difference any more between children and parents at the posttest due to an overall higher score for the group of parents. In more detail, the observed disparity stemmed from the difference between controls and parents of controls, while the children with dyslexia did not differ significantly from their parents at both test points. This fact may again indicate more serious concerns in children with dyslexia and their parents in everyday life.

The children`s verbal subjective reports about their experience learning Chinese characters agreed with the objective eye movement data and error rates.

### Limitations and strengths of the current study

One limitation of our study was the short training period. One could argue that the children of both groups were beginners and that a longer learning period might have disclosed a larger difference between the groups. On the other hand, a longer series of Chinese lessons than in the present study could provide more time for repetition so that more characters could be stored in long-term memory. Another limitation is the limited number of stimuli, which we accepted in order to prevent fatigue in the children by further extending the time spent in examination.

#### Strengths and new aspects

We examined children with isolated dyslexia without co-morbidities by applying strict inclusion criteria, which gave us a homogeneous sample and reduced the influence of other factors.

We developed and applied child-friendly Chinese lessons that were especially designed to avoid the use of alphabetic script.

Our study assessed for the first time the eye movement pattern during naming Chinese characters in German and Chinese, which showed the same performance in both groups for the correctly named characters. The difference became visible as a higher error rate in group DD, especially in the Chinese naming task.

Our results allow the conclusion that naming Chinese characters in German is mainly based on semantic processing, whereas naming them in Chinese requires an interaction between semantic and phonological processing.

We see an advantage to compare objective data with subjective reports and applied quality of life questionnaires before and after the lessons. QoL (LQ_0-28_) did not differ between groups from the children’s perspective at pretest. Parents of group DD perceived their children`s QoL to be lower compared with parents of group C at pre- and posttest.

Furthermore, the children`s verbal subjective reports about their learning experience agreed with the objective data.

## Conclusions and outlook

The current study has shown that children with dyslexia, when facing the task of naming Chinese character in German, were capable of utilizing the unimpaired visuo-spatial sketchpad of working memory to directly access the semantic system. However, in the task of naming characters in Chinese, their error rate increased markedly, while the task performed by the control group demonstrated that the use of the phonological loop was required. Future studies will have to clarify whether children with dyslexia could overcome or compensate for their deficits in naming Chinese characters by being subjected to a more extensive training.

## Supporting information

S1 Checklist TREND checklist for non-randomized controlled trials. (PDF)

S1 File Illustration of the stimuli used for the different tasks. Alphabetic words reading task, picture naming task, Chinese Character naming in German task and Chinese character naming in Chinese task.
(PDF)

S2 File Ethics protocol in German. The ethics protocol in the original German language.
(PDF)

S3 File Ethics protocol in English. The English version of the Ethics Protocol, translated from German.
(PDF)
