# Supplementary material for: Is learning a logographic script easier than reading an alphabetic script for German children with dyslexia?
Source: PLoS One. 2023 Feb 24;18(2):e0282200. doi: 10.1371/journal.pone.0282200 (PMC9956901; doi:10.1371/journal.pone.0282200)
Supplement: S1 File — Alphabetic words reading task, picture naming task, Chinese Character naming in German task and Chinese character naming in Chinese task. (PDF) [file pone.0282200.s002.pdf]

alphabetic words reading (W1-W8)

	German (English)	length	Frequency
W1	Hund (dog)	short	frequent
W2	Geige (violin)	short	rare
W3	Bohne (bean)	short	rare
W4	König (king)	short	frequent
W5	Gesicht (face)	long	frequent
W6	Bastrock (bast skirt)	long	rare
W7	Laterne (lantern)	long	rare
W8	Schularbeiten (schoolwork)	long	frequent

Alphabetic words w1-w8 of different word length (short, long) and frequency in the German language (frequent, rare) were presented in consecutive order. The font size was 14 ppt, Times New Roman (*1.6 fold*). At a viewing distance of 25cm the small letter “n” was of size 0.5°

picture naming (P1-P6)

	picture	German (English)
P1	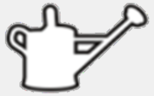	Gießkanne (watering can)
P2	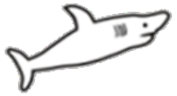	Hai (shark)
P3	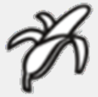	Banane (banana)
P4	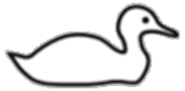	Ente (duck)
P5	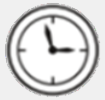	Uhr (clock)
P6	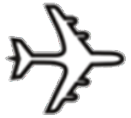	Flugzeug (airplane)

The size of the pictures differed in horizontal and vertical extent between 1.8° and $4,4^{\circ}$ (horizontal extent of “duck”). The original aspect ratio of each pictograms template was retained. The main criterion for the selection of the pictures was that they were easily recognizable and familiar to the children. For the picture “shark” and “duck”, also “fish” and “bird” were accepted as a correct answer.

Chinese characters, German naming task (G1-G12)

	char	German (English)
G1	人	Mensch (man, human)
G2	木	Baum (tree)
G3	龙	Drache (dragon)
G4	她	sie (she)
G5	属	gehören zu (belonging to)
G6	鼠	Ratte (rat)
G7	王	König (king)
G8	天	Himmel (sky)
G9	雨	Regen (rain)
G10	蛇	Schlange (snake)
G11	猫	Katze (cat)
G12	猪	Schwein (pig)

Chinese characters, German pronunciation (Characters No. G1-G6 with Infrared Eye Tracking, Characters No. G7-G12, with Scanning Laser Ophthalmoscope) each with increasing visual Complexity .

The Chinese characters were presented with the font SimHei in size 26pt, which corresponded to 1.8° at a viewing distance of 25cm. The size in degrees as well as its typeface, was determined in consultation with Chinese native speakers from the China Centrum Tübingen (CCT) and the Department of Quantitative Linguistics at the Faculty of Modern Languages, Tübingen: Tests were carried out on the screen with native Chinese colleagues. The smallest possible font size that could still be easily read was determined. The font should be kept simple and clear. The size of the pictures in the picture naming task has been carefully adapted to the size of the Chinese characters without changing the original aspect ratio of each picture’s template.

Chinese characters, Chinese naming task (C1-C12)

	char	Pinyin, German (English)
C1	大	dà, groß (big)
C2	石	shí, Stein (stone)
C3	我	wǒ, Ich (I)
C4	虎	hǔ, Tiger (tiger)
C5	猫	māo, Katze (cat)
C6	猪	zhū, Schwein (pig)
C7	山	shān, Berg (mountain)
C8	小	xiǎo, klein (small)
C9	鸡	jī, Hahn (rooster)
C10	他	tā, er (he)
C11	猴	hóu, Affe (monkey)
C12	鼠	shǔ, Ratte (rat)

Chinese characters, Chinese pronunciation (Characters No. C1-C6 with Infrared Eye Tracking, Characters No. C7-C12, with Scanning Laser Ophthalmoscope, SLO) each with increasing visual Complexity.

The Chinese characters were presented with the font SimHei in size 26pt, which corresponded to 1.8° at a viewing distance of 25cm. The size in degrees as well as its typeface, was determined in consultation with Chinese native speakers from the China Centrum Tübingen (CCT) and the Department of Quantitative Linguistics at the Faculty of Modern Languages, Tübingen: Tests were carried out on the screen with native Chinese colleagues. The smallest possible font size that could still be easily read was determined. The font should be kept simple and clear. The size of the pictures in the picture naming task has been carefully adapted to the size of the Chinese characters without changing the original aspect ratio of each pictogram’s template.
