# Supplementary material for: Is learning a logographic script easier than reading an alphabetic script for German children with dyslexia?
Source: PLoS One. 2023 Feb 24;18(2):e0282200. doi: 10.1371/journal.pone.0282200 (PMC9956901; doi:10.1371/journal.pone.0282200)
Supplement: S2 File — The ethics protocol in the original German language. (PDF) [file pone.0282200.s003.pdf]

497/2018B01 vom 2.7.18 Überarbeitete Version vom 16.10.18

Antrag zur Beurteilung ethischer und rechtlicher Fragen eines medizinischen Forschungsvorhabens am Menschen: Prüfplan

TITEL

Erfassung und Förderung visueller Fähigkeiten bei deutschsprachigen Kindern mit und ohne Legasthenie durch Erlernen chinesischer Schriftzeichen.
Visuelle Verarbeitung und pädagogische Anwendung

ANTRAGSTELLERIN:
Prof. Dr. med. Susanne Trauzettel-Klosinski
Tel: 07071-298-0818, Fax: 07071-29-5164,
E-Mail: susanne.trauzettel-klosinski@uni-tuebingen.de

Forschungsstelle:
Forschungseinheit für Visuelle Rehabilitation
Department für Augenheilkunde, Universität Tübingen
Elfriede Aulhorn Str. 7, 72076 Tübingen

Arbeitsgruppe:

Augenklinik Tübingen, Forschungseinheit für Visuelle Rehabilitation

- Prof. Dr. med. Susanne Trauzettel-Klosinski, Leiterin der Forschungseinheit für Visuelle Rehabilitation, Department für Augenheilkunde-Tübingen; Leiterin, Supervisorin und Koordinatorin des Projekts
- Wissenschaftlicher Mitarbeiter Dr.med. Dipl. inf. Stephan Küster
- Angelika Cordey, Orthoptistin
- Theda Faisst, Doktorandin
- Maria Jose Galvez de Villalta, Projektmanagement

KOOPERATIONEN

1. MEG- Zentrum am Universitätsklinikum Tübingen: Prof. Dr. Christoph Braun
Es besteht bereits eine langjährige Kooperation mit mehreren gemeinsamen Publikationen.
2. China Centrum Tübingen (CCT):
Prof. Dr. Helwig Schmidt-Glintzer, Direktor des CCT und
Präsident des Erich-Paulun-Instituts, Universität Tübingen, Seniorprofessor
Das Institut wendet sich unter Nutzung eines Netzwerks aus Kultur, Wirtschaft, Wissenschaft und Politik an Schüler und Studenten, die die chinesische Sprache erlernen. Dr. Vera Schick
CCT, Erich-Paulun-Institut, Leiterin EPI-Programm, Staatliches Seminar für Didaktik und Lehrerbildung (Gymnasien) Tübingen, Fachleiterin Chinesisch
3. Seminar für Sprachwissenschaft Universität Tübingen:
Prof. Dr. H. Baayen, Leiter der Forschungseinheit „Quantitative Linguistics“ Dr. Chin-Chu Sun (Post-Doktorandin und native Chinese speaker)
4.Geschwister Scholl Gemeinschaftsschule Tübingen (Rektor C. Schnittert) zur Rekrutierung der normal-lesenden Kinder und einem Teil der Legastheniker

Antragsart : Überarbeitete Version

Für alle hier verwendeten Untersuchungsmethoden liegen Ethikvoten zu früheren Projekten vor.
Für die klinischen Untersuchungsmethoden: 325/2009B01.
Eye Tracking bei Kindern: 227/2014BO1, für SLO-und MEG-Studien 252/2007BO1.

FINANZIERUNG:

Das Projekt wird von der Exzellenzinitiative der Universität Tübingen finanziert.
Außerdem wird das Projekt über die Charlotte und Tistou Kerstan-Stiftung und die Werner Kossmann Stiftung unterstützt

Inhaltsverzeichnis:

1. Zusammenfassung
2. Einführung
    2.1 Stand der Forschung
    2.2 Eigene Vorarbeiten
3. Studienziele
4. Studienpopulation
    4.1. Begründung für die Durchführung der Studie bei Minderjährigen
    4.2. Rekrutierung
    4.3. Ein- und Ausschlusskriterien
    4.4. Studienmedikation
5. Studienablauf und Untersuchungsmethoden
    5.1. Studiendesigns
    5.2. Durchführung
    5.3. Zeitplan
    5.4. Untersuchungsmethoden
6. Abbruchkriterien
7. Risiken und Nebenwirkung- Klinische und wissenschaftliche Bewertung
8. Angaben zur statistischen Auswertung
9. Klinische und wissenschaftliche Bewertung
10. Versicherung
11. Datenschutz
12. Aufklärung der Studienteilnehmer
13. Kooperationen
14. Literatur
15. Anlagen

1. ZUSAMMENFASSUNG

Das Projekt ist innovativ in seiner Fragestellung und hat folgende Schwerpunkte:
Einen grundlagenorientierten Teil zur visuellen und kortikalen Verarbeitung chinesischer Schriftzeichen sowie einen anwendungsorientierten sprachwissenschaftlichen und pädagogischen Teil von gesellschaftlicher Relevanz.
Es handelt sich somit um ein interdisziplinäres Projekt und umfasst folgende Fachgebiete: Augenheilkunde, Neuro-Ophthalmologie, Neuro-Sensorik, Sinologie, Linguistik, Pädagogik

Die Legasthenie ist eine häufige Entwicklungsstörung des Lesens und Schreibens (4-5% der deutschen Schulkinder). Die Betroffenen sind in der Schule und im Berufsleben stark benachteiligt, sodass es oft zu psychischen und sozialen Folgeschäden kommt.
In der Forschung wurden bisher vorwiegend die Defizite der Kinder untersucht, während wenig auf deren Stärken, insbesondere ihre visuellen Fähigkeiten, geachtet wurde.
In unseren Untersuchungen hat sich gezeigt, dass deutschsprachige legasthenische Kinder nur bei den schriftsprachlichen Stimuli Probleme hatten, während bildliches Material (Piktogramme) gleich gut wie bei Nicht-Legasthenikern verarbeitet wurde. Es soll deshalb erstmalig untersucht werden, ob deutschsprachige legasthenische Kinder die nicht buchstaben-gebundene logographische chinesische Schrift genauso gut erlernen können wie ihre normal-lesenden Klassenkameraden, und wie die Schriftzeichen visuell verarbeitet werden.
Es sollen je 24 Kinder aus der 4. und 5. Klasse mit und ohne Legasthenie in die Studie aufgenommen werden, davon je 3 in die Pilotstudie.
Eine genaue Analyse der Fixationen auf den Schriftzeichen mittels eines Scanning Laser Ophthalmoskops, mit dem man den Lesevorgang direkt auf der Netzhaut erfassen kann, soll wichtige Hinweise auf die Art der Verarbeitung (lokal oder global) geben. Außerdem soll mittels Magnetenzephalographie die kortikale Weiterverarbeitung untersucht werden.
Die Kinder erhalten dann einen 24-stündigen Blockunterricht in Chinesisch, wofür das Unterrichtsmaterial zuvor kindgerecht entwickelt wird. Dabei ist von Interesse, wie in beiden Gruppen die Schriftzeichen erlernt werden und ob die Legastheniker davon profitieren.
Wenn dies der Fall wäre, könnten legasthenische Kinder durch die speziellen Sprachkenntnisse einen Vorteil in Schule und Beruf erlangen und müssten nicht wie bisher die „ewigen Verlierer“ sein. Sie könnten damit auch ihr Selbstvertrauen und ihre psychische und soziale Entwicklung positiv beeinflussen.
Wenn sich bei den normal-lesenden Kindern zeigt, dass sie die chinesischen Schriftzeichen in der 4. und 5. Klasse lernen können, könnten sich daraus Empfehlungen für Chinesisch-Unterricht in den weiterführenden Schulen ergeben.
Im Hinblick auf die zunehmende Bedeutung wirtschaftlicher und kultureller Beziehungen zu China wäre diese Kompetenz auch bildungspolitisch und gesellschaftlich von Nutzen.

2. EINFÜHRUNG

2.1. Stand der Forschung

Bei der Lese-Rechtschreibstörung (LRS, Legasthenie) handelt es sich um eine umschriebene Entwicklungsstörung des Lesens und Schreibens. Von einer LRS sind im deutschsprachigen Raum ca. 4-5 % der Schüler betroffen. Die Kinder sind in ihrer schulischen und beruflichen

Laufbahn trotz Fördermaßnahmen stark beeinträchtigt und entwickeln oft psychische und soziale Folgeschäden, weil sie sich stets als Verlierer fühlen. Etwa 30% der Legastheniker werden delinquent. (Übersichtsarbeiten siehe Mayer 2016, Schulte-Körne 2007, Warnke et al 2002). Eine allgemein anerkannte Entstehungsursache der Legasthenie in alphabetischen Sprachen beruht auf der Schwierigkeit, Buchstaben in Laute umzuwandeln (phonologisches Defizit). In eigenen Untersuchungen konnten wir zeigen, dass legasthenische Kinder nur bei buchstabengebundenen Aufgaben Schwierigkeiten hatten, aber beim Benennen von
Piktogrammen (ohne Zeitdruck) gleich gut wie leseungestörte Kinder waren (Trauzettel-Klosinski et al 2002).
Während die Forschung bisher vorwiegend auf die Defizite dieser Kinder fokussiert hat, wurde wenig zu deren Stärken und der Förderung derselben untersucht. Das potenzielle visuelle Talent der Kinder wurde bisher nicht genügend beachtet.
Über logographische Sprachen hat sich erst in den letzten Jahren ein intensives Forschungsfeld entwickelt. Neuere Untersuchungen haben gezeigt, dass logographische Schriften wie Chinesisch primär visuell entschlüsselt werden (Hua et al 2006, Wang LC 2016). Deshalb basiert eine Legasthenie bei chinesischen Kindern vorwiegend auf einem Defizit in der visuellen Verarbeitung der Schriftzeichen (Zhao et al 2014, Quian et al 2015 und 2016, Liu et al 2012, Meng et al 2011 und 2014, Wang et al 2010). Berichte über phonologische Defizite bei chinesischen
Legasthenikern (Cao et al 2017, Siok et al 2009) könnten möglicherweise auf Untergruppen mit unterschiedlichen oder kombinierten Defiziten hinweisen.
Die Verarbeitung der Reize (Buchstaben versus Piktogramme) im Gehirn erfolgt in unterschiedlichen Hirnarealen, wie wir in eigenen Untersuchungen (Trauzettel-Klosinski et al 2006) fanden (s.u.). Zur Verarbeitung chinesischer im Vergleich zu alphabetischer Schrift bei
chinesischen Lesern ohne Lesestörung gibt es einige Studien mit funktioneller Kernspintomographie (fMRI) und event-related Potenzialen (ERPs), die eine teilweise Übereinstimmung der aktivierten Hirnareale, jedoch auch zusätzliche Aktivitäten beschrieben haben, vor allem in der rechten Hirnhälfte, in der die visuell-räumliche Verarbeitung stattfindet (Hsu et al 2011, Wu et al 2012). Bei normal-lesenden chinesischen Probanden wurden auch
Effekte der visuellen Komplexität (Hsu et al 2011), der Häufigkeit eines Schriftzeichens (Kuo et al 2003, Lee et al 2004) sowie der orthographischen Konsistenz (Lee et al 2004) und der homophonen Dichte (Chen et al 2016) beschrieben.

2.2 Eigene Vorarbeiten

Messung der Augenbewegungen während des Lesens

Mit einem Scanning Laser Ophthalmoskop (SLO) können simultan die Netzhaut und die Stimuli (Text oder Piktogramme) abgebildet werden und das Abscannen der Zeilen „live“ auf der Netzhaut betrachtet werden (Abbildung 1).
Bei der Registrierung der Augenbewegungen während des Lesens machen Legastheniker zahlreiche Blicksprünge in Leserichtung (Sakkaden) und rückwärts (Regressionen) und haben eine stark verlangsamte Lesegeschwindigkeit (Mackeben et al 2004, Trauzettel-Klosinski et al
2010). Außerdem konnten wir zeigen, dass die Leseproblematik mit zunehmendem phonologischem Schwierigkeitsgrad in der deutschen Sprache bei legasthenischen Kindern zunimmt (Dürrwächer et al 2010). Die Augenbewegungen können somit als Indikator für das phonologische Defizit dienen.

Ferner haben wir anhand von magnet-enzephalographischen (MEG)-Untersuchungen gezeigt, dass legasthenische Kinder beim Erlernen einer neuen alphabetischen (griechisch) Schrift im Vergleich zu einer normal-lesenden Kontrollgruppe keinen Vorteil dadurch hatten, dass beide Gruppen am „Nullpunkt“ begonnen haben, sondern genauso stark beeinträchtigt waren wie bei der lateinischen Schrift (Braun et al 2009). Somit haben wir in unseren eigenen untersuchten
Kollektiven das phonologische Defizit der deutschsprachigen Leser in der alphabetischen Sprache mit verschiedenen Methoden nachgewiesen.

Im Gegensatz dazu konnten die legasthenischen Kinder Piktogramme genauso schnell wie normal lesende Kinder benennen (Trauzettel-Klosinski et al 2002 b, Abbildung 1). Die von uns verwendete Aufgabe bestand in einem Benennen ohne Zeitdruck und ohne serielle Darbietung der bildlichen Stimuli. Sie unterscheidet sich somit von einer „rapid naming (RAN)“ - Aufgabe, bei der in der Literatur Defizite beschrieben wurden (Denkla & Rudel 1976, Swan & Goswami 1997, Wolf & Obregon 1992)

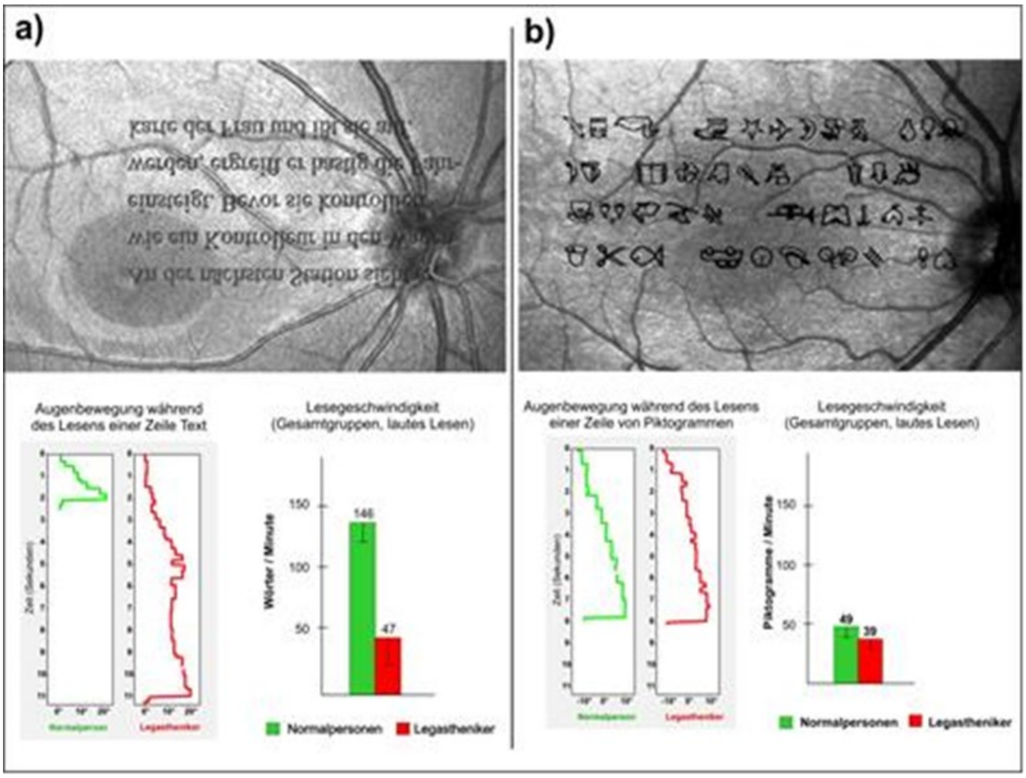

Abb 1: Messung des Lesevorgangs mittels SLO: Die Stimluli (a: Text, b: Piktogramme, angeordnet wie Text) sind simultan mit der Netzhaut sichtbar. Sie stehen nur für den Untersucher auf dem Kopf, für den Probanden aufrecht. a: beim Lesen von buchstaben-gebundener Information ist bei Legasthenikern die Lesegeschwindigkeit hochgradig
herabgesetzt und die Anzahl der Blicksprünge stark erhöht. Im Gegensatz dazu sind Legastheniker beim Benennen von Piktogrammen nicht beeinträchtigt (b).

Verarbeitung der Reize im Gehirn

Die kortikale Verarbeitung der Reize (Buchstaben versus Piktogramme) erfolgt in unterschiedlichen Hirnarealen, wie wir in eigenen Untersuchungen (Trauzettel-Klosinski et al 2006) fanden. Es zeigten sich auch hier bei den Legasthenikern normale Reaktionszeiten für die Piktogramme und verlängerte Reaktionszeiten beim Lesen von Wörtern (Abb 2).

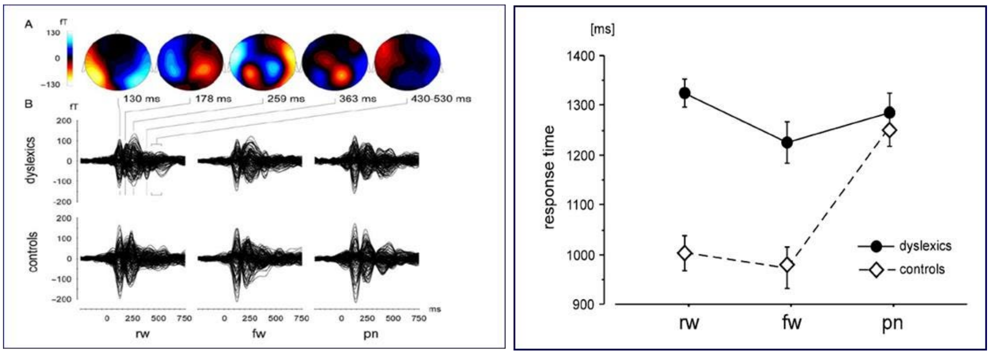

Abbildung 2:

Magnetische Aktivität während des Lesens von Wörtern und des Benennens von Piktogrammen am Magnetenzephalogramm (MEG): Bei der Piktogramm-Aufgabe besteht kein Unterschied zwischen den Gruppen, während beim Lesen von Wörtern die Legastheniker verzögerte Antworten zeigen im Vergleich zur Kontrollgruppe. rw: rare words, fw: frequent words, pn: pictograms (aus Trauzettel-Klosinski et al 2006)

Daraus ergab sich der Ansatz, dass legasthenische Kinder möglicherweise einen visuellen Vorteil haben könnten, wenn sie piktographische/logographische anstatt phonologische Aufgaben erhalten. Daraus ergab sich der Plan, dass Kinder, die in der alphabetischen Sprache legasthenisch sind, eine logographische Sprache erlernen sollten, um hier eventuell gleich gut wie ihre Klassenkameraden sein zu können.

Entwicklung standardisierter Lesetexte in 17 Sprachen:

Im Rahmen eines EU-Projekts (AMD-READ, QLK6-CT-2002-00214, Hahn et al 2006) und später weltweit wurden unter Koordination von Frau Prof. Trauzettel-Klosinski standardisierte Lesetexte mit Textabschnitten (nicht nur Einzelsätze) zur Messung der Lesegeschwindigkeit in 17 Sprachen entwickelt (Trauzettel-Klosinski et al 2012, www.precision-vision.com). Es stehen je 10 Texte pro Sprache zur Verfügung, die sich im Schwierigkeitsgrad und der linguistischen Komplexität nicht unterscheiden, sodass sie für wiederholte Messungen sehr gut geeignet sind. Die Entwicklung in den anderen Sprachen erfolgte nach demselben Prinzip (gleicher Inhalt, gleicher Schwierigkeitsgrad, gleiche linguistische Komplexität) und wurde von muttersprachlichen Linguisten erarbeitet. Auch Chinesisch steht zur Verfügung. Damit ist es erstmals möglich, internationale Lesestudien über die Sprachgrenzen hinweg durchzuführen.

3. FRAGESTELLUNGEN UND STUDIENZIELE

3.1. Fragestellungen

Aufgrund der o.g. Forschungsergebnisse ergeben sich theoretische und praktische Fragestellungen:

1) Werden piktographische/logographische Zeichen lokal (Analyse der Einzelkomponenten) oder global (als Gesamtobjekt) verarbeitet? Es gibt Hinweise aus der Literatur, dass chinesische Schriftzeichen vorwiegend global verarbeitet werden, also das Zeichen als Ganzes und nicht die einzelnen Striche verarbeitet werden.

2) Können deutschsprachigen legasthenische Kinder ihre visuelle Kompetenz, die sie möglicherweise bereits als Kompensationsstrategie geübt haben, nutzen, wenn sie Chinesisch lernen? Die chinesischen Zeichen sind logographisch und erfordern primär keine phonologische Analyse. In einer früheren Studie (Rozin et al 1971) wurde gezeigt, dass legasthenische Kinder ein Set von 30 chinesischen Zeichen innerhalb weniger Stunden lernen konnten, obwohl sie für Englisch eine hochgradige Legasthenie hatten. Allerdings durften die Kinder die Wörter auf
Englisch aussprechen. Die Frage ist nun, ob legasthenische Kinder und Jugendliche durch echten Chinesisch-Unterricht eine Förderung erhalten könnten, die ihren visuellen Stärken Rechnung trägt und ihnen neues Selbstvertrauen sowie einen Wettbewerbsvorteil in ihrer beruflichen Laufbahn verschaffen könnte.

3.2. Studienziele

1.Visuelle Informationsverarbeitung:

Die Untersuchung der Augenbewegungen soll folgende Aspekte erfassen:

- Unterschiede zwischen logographischer (chinesisch) und alphabetischer (deutsch) Schrift
- Bei den chinesischen Schriftzeichen:

o Lokale oder globale Verarbeitung
o Abhängigkeit von der visuellen Komplexität (siehe Tab. 1)
o Abhängigkeit vom phonologischen Anteil im Schriftzeichen

Damit soll untersucht werden, ob deutschsprachige legasthenische Kinder die chinesischen Schriftzeichen besser lernen können als die alphabetischen. Die Ergebnisse sollen zu einem besseren Verständnis der Ursachen der Legasthenie für alphabetische Schrift und der damit assoziierten Verarbeitungsstörungen im Gehirn sowie den Möglichkeiten einer Förderung über das visuelle System beitragen.

2.Kortikale Informationsverarbeitung:

Es ist anzunehmen, dass die Schriftzeichen unterschiedlich im Gehirn weiterverarbeitet werden. Eventuell können Defizite in den Spracharealen für alphabetisches Lesen durch gute Funktionen in Spracharealen für logographische Schrift kompensiert werden. Die Untersuchungen sollen zu einem besseren Verständnis der neuronalen Netzwerke, die der Verarbeitung der chinesischen Schriftzeichen zugrunde liegen, beitragen.

3.Erlernen der chinesischen Schrift und Umsetzung in die Praxis

Kindgerechtes Unterrichtsmaterial soll entwickelt und dann in einem Unterrichtsblock eingesetzt und evaluiert werden.

4.Bildungspolitische Auswirkungen:

- Wenn die nicht-legasthenischen Kinder bereits in der 4. und 5. Klasse Chinesisch lernen können, wäre ein Chinesisch-Unterricht in den weiterführenden Schulen eine Option, da die Bedeutung der chinesischen Sprache und Kultur zunehmen wird.
- Wenn die legasthenischen Kinder Chinesisch lernen können, wäre das Angebot eines Chinesisch-Unterrichts in den Schulen eine Möglichkeit, diese Kinder über ihre visuellen Fähigkeiten

zu fördern (was natürlich nicht die Legasthenie-spezifische Förderung für die alphabetische Sprache ersetzen soll!).

5.Psychische und soziale Auswirkungen:

Legasthenische Kinder könnten durch das Erlernen des Chinesischen einen Wettbewerbsvorteil in Schule und Beruf erlangen und müssten nicht wie bisher die „ewigen Verlierer“ sein. Sie könnten damit auch ihr Selbstvertrauen und ihre psychische und soziale Entwicklung positiv beeinflussen. Folgeschäden könnte damit vorgebeugt werden.

6.Gesellschaftliche Bedeutung:

In Anbetracht der zunehmenden Bedeutung des kulturellen, wirtschaftlichen und wissenschaftlichen Austausches mit China wäre es auch gesellschaftlich von Vorteil, wenn mehr deutschsprachige Kinder die chinesische Sprache erlernen könnten.

4. STUDIENPOPULATION

4.1. Begründung, warum die Studie an Minderjährigen durchgeführt werden muss:

Eine Lese-Rechtschreibstörung wird in der Regel in der 3.-4. Klasse, manchmal auch erst in der 5. Klasse diagnostiziert. Kinder nehmen ihre Welt vorwiegend visuell wahr und haben im Vergleich zu Erwachsenen ein besonders gutes visuelles Gedächtnis, was für das Lernen und Erinnern von chinesischen Schriftzeichen ein Vorteil sein kann. Außerdem können Kinder umso besser eine neue Sprache lernen, je jünger sie sind. Das Erlernen der chinesischen Sprache in der 4. und 5. Klasse, wenn also ein Übertritt in weiterführende Schulen stattfindet, ist dafür ein besonders günstiger Zeitpunkt.

4.2 Rekrutierung

Die Rekrutierung der legasthenischen Kinder erfolgt über den Bundes- und Landesverband Legasthenie sowie über Legasthenie-Diagnostik- und Therapieeinrichtungen im Raum Tübingen.

Die Rekrutierung der Kontrollkinder erfolgt vorwiegend über die Geschwister Scholl Gemeinschaftsschule Tübingen, außerdem über eine Rundmail an der Universität Tübingen.

Auf die Freiwilligkeit der Teilnahme wird besonders hingewiesen.

4.3. Ein- und Ausschlusskriterien

Einschlusskriterien:

- Kinder mit und ohne Lese-Rechtschreibstörung der 4. und 5. Klasse
- gesicherte Diagnose Legasthenie (mit standardisierten Testverfahren)
- Bereitschaft, alle Studientermine wahrzunehmen

Ausschlusskriterien

- Ko-Morbiditäten, wie z. B. ADHS
- Augenerkrankungen außer Refraktionsanomalien

4.4. Studienmedikation

In der Studie werden keine Medikamente getestet. Eine Erweiterung der Pupille und eine Zykloplegie kann bei einigen Kindern notwendig sein (siehe Kapitel 7).

5. STUDIENABLAUF UND UNTERSUCHUNGSMETHODEN

5.1 Studiendesign

Die geplante Studie ist eine prospektive klinische und pädagogische Diagnostik- und Interventionsstudie mit legasthenischen und normal-lesenden Kindern, die im Herbst 2018 in die 5. Klasse kommen.

Pilotstudie: Je 3 Kinder mit und ohne Legasthenie
Hauptstudie: Je 21 Kinder der 4. und 5. Klasse mit und ohne Legasthenie

1) Augenärztliche Voruntersuchung zum Ausschluss visueller Defizite
2) Untersuchung der Augenbewegungen beim Lesen von alphabetischem Text und bekannten Piktogrammen am SLO und Eye Tracker
3) Untersuchung der Weiterverarbeitung der Stimuli im Gehirn mittels MEG
4) Blockunterricht der Chinesischen Sprache 2 Wochen in den Schulferien (24 Schulstunden, 6-8 Kinder pro Lerngruppe)
5) Kontroll-Untersuchung der Augenbewegungen beim Lesen von alphabetischem Text und den neu gelernten chinesischen Zeichen am SLO und Eye Tracker
6) Untersuchung der Weiterverarbeitung der Stimuli im Gehirn mittels MEG

5.2 Durchführung

Die Augen-Untersuchungen am werden in der Forschungseinheit für Visuelle Rehabilitation des Departments für Augenheilkunde der Universität Tübingen, Elfriede Aulhorn Str. 7, durchgeführt,
die MEG-Untersuchungen am MEG-Zentrum des UKT, Otfried Müller Strasse.
Der Chinesisch-Unterricht findet am China-Zentrum Tübingen, Hintere Grabenstrasse, statt.

5.3 Zeitplan

<u>Monat 1-3: Vorbereitung</u>
Genaue Entwicklung des Studiendesigns, Entwicklung der Stimuli: Auswahl geeigneter chinesischer Zeichen in Abstimmung mit den Kooperationspartnern (neu erschienene Datenbank, Sun 2016). Vorbereitung des Unterrichtsmaterials, Ethikantrag, Rekrutierung von legasthenischen und normal-lesenden Kindern der 4. und 5. Klasse
<u>Monat 4-6: Pilotstudie mit je 3 Kindern mit und ohne Legasthenie</u>
Erprobung und Optimierung der Stimuli, Erprobung und Optimierung des Unterrichtsmaterials und der Durchführung des Unterrichts. 1.Unterrichtsblock. Rekrutierung für die Hauptstudie
<u>Monat 7-12: Beginn der Hauptstudie:</u> Datenerhebung und Unterricht
<u>Monat 13-18</u>: Fortsetzung der Datenerhebung, Unterricht, Beginn der Auswertung
<u>Monat 19-21:</u> Gesamtauswertung aller Daten, sehr zeitaufwändig
<u>Monat 22-24</u>: Verfassen der Publikationen, Öffentlichkeitsarbeit, Transfer in die Praxis

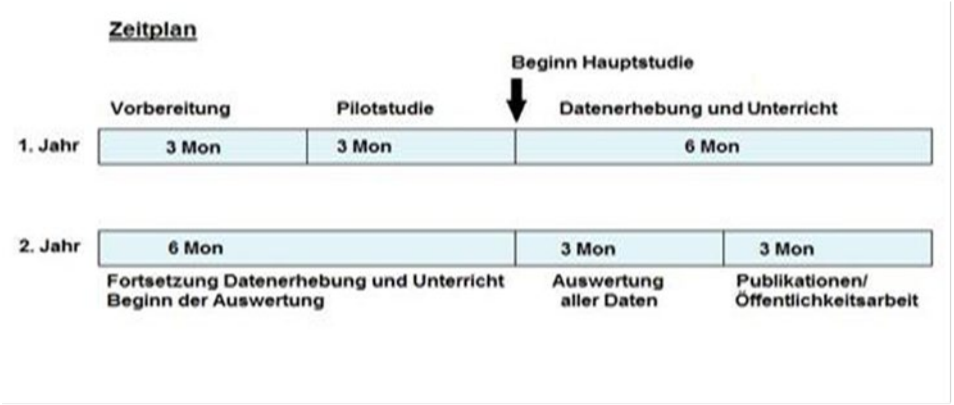

5.4. Untersuchungsmethoden

Alle Untersuchungen werden ambulant durchgeführt. Die Erstuntersuchung in der Augenklinik dauert ca. 1,5- 2 Stunden pro Patient mit zusätzlichen Pausen. Die Kontrolluntersuchung dauert 0,5 -1 Stunde. Die Untersuchungen am MEG finden i.d.R. an einem anderen Tag statt und sind mit ca. einer halben Stunde angesetzt.

5.4.1. Augenärztlich-orthoptische Voruntersuchung

- Ermittlung der Sehschärfe für die Ferne und für die Nähe bei standardisiertem Beleuchtungsniveau. Einbezogen ist die objektive Refraktionsbestimmung mittels Skiaskopie oder Refraktometer.
- Orthoptischer Status: Fixation, Augenstellung, Motilität, beidäugiges Sehen, Dominanz
- Klinisch-morphologische Untersuchung der vorderen und hinteren Augenabschnitte mit genauer Erhebung des ophthalmologischen Befundes.

5.4.2. Fragebögen zur Lebensqualität

Zu Beginn der Studie wird ein standardisierter Fragebogen zur Lebensqualität eingesetzt

5.4.3. Messung der Augenbewegungen

Augenbewegungen während des Lesens von deutschem und chinesischem Text sowie beim Bennen von Piktogrammen werden mittels eines Infrarot Eye Trackers (Saccadometer, Jazz novo, Ober, Abbildung 3). Die Messeinheit befindet sich an der Nasenwurzel und beleuchtet tangential den Limbus. Sie wird mit einem elastischen Band am Kopf befestigt. Das System hat damit vor allem für Kinder den Vorteil, nicht direkt vor den Augen zu sitzen. Die Methode haben wir bereits in einer soeben abgeschlossenen Studie bei Kindern eingesetzt (Ethik-Votum: 227/2014BO1).

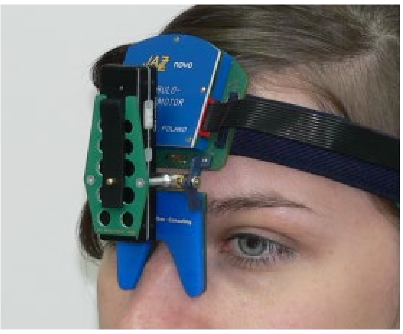

Abbildung 3: Infrarot Limbus Eye tracker

5.4.4. Untersuchung der visuellen Verarbeitung der Stimuli (alphabetisch und pictographisch) stehen folgende Methoden zur Verfügung:

Auf der Netzhaut

Mit einem Scanning Laser Ophthalmoskop (SLO) können simultan die Netzhaut und die dargebotenen Zeichen dargestellt und genau erfasst werden, wie ein Zeichen abgescannt wird (siehe Abb. 1). Man sieht also ein live-Bild des Lesevorgangs direkt auf der Netzhaut. Mit der gezielten Auswahl der chinesischen Zeichen sollen mittels der Fixationen auf dem Zeichen folgende Fragen untersucht werden:

- Lokale (Fixation der einzelnen Striche) oder globale (als Gesamtobjekt) Verarbeitung
- Abhängigkeit von der visuellen Komplexität (Tabelle 1)
- Einfluss des ikonographischen Abstraktionsgrades

Tabelle 1: Beispiele geringer und hoher visueller Komplexität (nach Sun 2016). Mit zunehmender Anzahl der Striche wird das Zeichen komplexer.

Character	Stroke	Meaning
个	3	each
露	21	expose

Kortikale Weiterverarbeitung

Ferner kann die Verarbeitung der Reize im Gehirn mittels Magneto-Enzephalographie MEG mit hoher zeitlicher Auflösung (Millisekunden) aufgezeichnet werden (in Kooperation mit dem MEG-Zentrum) (siehe Abb. 2). Es handelt sich dabei – wie auch beim SLO - um eine vollkommen non-invasive Methode, die wir auch schon in früheren Studien bei Kindern eingesetzt haben.

5.4.5. Chinesisch-Unterricht

Zunächst wird das Unterrichtsmaterial von unserem Kooperationspartner am CCT kindgerecht entwickelt. Dann erhalten die Kinder (Legastheniker und Kontrollen) Blockunterricht während der Schulferien: In kleinen Gruppen à 6-8 Schülern findet an 8 Ferientagen jeweils ein 3-stündiger Unterricht statt mit einer Pause. Somit erhält jedes Kind insgesamt 24 Schulstunden (à 45 Minuten) Unterricht.

6. ABBRUCHKRITERIEN

Die Studienteilnahme kann von den Patienten jederzeit ohne Angabe von Gründen folgenlos abgebrochen werden.

7. RISIKEN UND NEBENWIRKUNGEN

Die im Rahmen der Studie durchgeführten ophthalmologischen Untersuchungen entsprechen hinsichtlich Risiken und möglicher Nebenwirkungen denen einer augenärztlichen Routineuntersuchung.

Für die Untersuchung des Augenhintergrundes ist gelegentlich eine Weitstellung der Pupillen mit einem kombinierten Parasympatholytikum und Sympathomimetikum erforderlich. Zur Pupillenerweiterung verwenden wir das parasympatholytische Medikament Tropicamid topisch, das auch in der Routine augenärztlicher Untersuchungen eingesetzt wird. Die Gefahr eines Glaukomanfalls durch die Pupillenerweiterung ist bei Kindern kaum vorhanden.

Zur objektiven Refraktionsbestimmung ist bei jüngeren Kindern manchmal eine Cycloplegie erforderlich. Dazu verabreichen wir Augentropfen, die den Wirkstoff Cyclopentolathydrochlorid (Cyclopentolat 1%, oder 0,5%) enthalten und i.d.R. 2-3x im Abstand von 10 Minuten getropft werden. Die Pupillenerweiterung hält für ca. 3-4 Stunden an. Während dieser Zeit, kann es zu einer verstärkten Lichtempfindlichkeit und einer Einschränkung des Sehvermögens in der Nähe kommen.
Der hier verwendete Infrarot Eye Tracker ist besonders gut für Kinder geeignet, da er nur mit einem Kopfband befestigt ist und die Messeinheit nicht direkt vor den Augen sitzt.

Das Scanning Laser Ophthalmoskop (SLO) tastet mit einem schwachen Laserstrahl die Netzhaut ab. Das Gerät besitzt zwei unabhängig voneinander arbeitende Sicherheitssysteme und erfüllt die Anforderungen der schwächsten Laser-Sicherheitsklasse I, d.h., dass zu keinem Zeitpunkt schädliche Laserstrahlung auf Ihr Auge einwirken kann. Der Laser hat eine rein diagnostische Funktion.
Das SLO erlaubt die simultane Erfassung des Netzhautbildes und des Stimulus und liefert deshalb die absolute Position der Fovea auf dem Stimulus. Sie erfordert bei den meisten Patienten eine Pupillenerweiterung. Die Methode wird von uns seit vielen Jahren zur Leseanalyse eingesetzt (Ethik-Voten 077/2012 BO1, 176/2003V).

8. KLINISCHE UND WISSENSCHAFTLICHE BEWERTUNG

Die Studie wird wichtige theoretische Erkenntnisse zur Informationsverarbeitung von buchstabengebundener versus bildlicher (logographischer) Schrift erbringen. Für die Verarbeitung chinesischer Schriftzeichen werden wir wichtige Informationen erhalten bezüglich lokaler oder globaler Verarbeitung sowie der Abhängigkeit von der visuellen Komplexität und dem phonologischen Anteil.
Die Anwendung besteht in konkretem Chinesisch-Unterricht. Wenn die normal-lesenden Kinder die chinesischen Zeichen lernen können, wäre ein solches Angebot in den weiterführenden Schulen eine Option. Den legasthenischen Kindern böte es die Möglichkeit, ihre visuellen Stärken zu nützen und durch diese Sprachkenntnisse eine positive Bilanz in ihrer schulischen und auch beruflichen Laufbahn aufweisen zu können. Dies wäre auch von gesellschaftlicher Relevanz, wenn man die zunehmende Bedeutung der wirtschaftlichen, kulturellen und wissenschaftlichen Beziehungen mit China bedenkt.

9. ANGABEN ZUR STATISTISCHEN AUSWERTUNG

Es ist geplant, die Daten mittels einer Varianzanalyse auszuwerten und bei Nicht- Normalverteilung non-parametrische Tests verwenden.

10. Versicherung

Für den Weg zu oder von den Untersuchungsterminen sowie für den Unterricht wird eine Wege- und Aufenthaltsversicherung abgeschlossen. Wege-Unfall-Versicherung Nr. 50073896666, Versicherer: SV SparkassenVersicherung AG

11. Datenschutz

Die Daten werden auf Fragebögen bzw. Untersuchungsbögen und in Computerdateien erfasst. Die Daten werden direkt nach der Erfassung in pseudonymisierter Form gespeichert. Dazu wird ein Code zugeteilt und eine Liste geführt, die eine Rückführung der Daten ermöglichen würde.
Die Kodierungsunterlagen und die Kodierungsliste werden zusammen mit den Einverständniserklärungen in einem verschlossenen Schrank aufbewahrt, zu dem nur die Prüfärzte

Zugang haben. Sie wird Dritten nur in den in der Einwilligungserklärung zum Datenschutz genannten Fällen zugänglich gemacht. Die Daten werden 10 Jahre aufbewahrt.

Gesondertes Informationsblatt des UKT wird beigelegt.

12. Aufklärung der Studienteilnehmer

Jeder Proband und jeder Patient/in mit zumindest einem Elternteil werden vor ihrer Einwilligung durch den Untersuchungsleiter ausführlich über den Ablauf der Untersuchung informiert. Alle Probanden/Patienten und deren Eltern werden ausdrücklich darauf hingewiesen, dass sie ihre Teilnahme, bzw. die Teilnahme ihres Kindes, an der Studie jederzeit und ohne Angabe von Gründen beenden können. Erst danach geben die Untersuchungsteilnehmer und deren Eltern ihre Zustimmung zu den Untersuchungen.
Vergütung: Die Kinder erhalten nach Abschluss der letzten Untersuchung einen Gutschein (Bücher, Kinobesuch) im Wert von 25.-€, die Eltern erhalten zusätzlich eine Aufwandsentschädigung von 25.-€.

13. Kooperationen (Einzelheiten siehe Seite 1)

Tübingen, den 16.10..2018

Prof. Dr. med. Susanne Trauzettel-Klosinski

15. Anlagen: Aufklärungs- und Einwilligungsblatt
