# Supplementary material for: Is learning a logographic script easier than reading an alphabetic script for German children with dyslexia?
Source: PLoS One. 2023 Feb 24;18(2):e0282200. doi: 10.1371/journal.pone.0282200 (PMC9956901; doi:10.1371/journal.pone.0282200)
Supplement: S3 File — The English version of the Ethics Protocol, translated from German. (PDF) [file pone.0282200.s004.pdf]

497/2018B01 of 2.7.18 Revised version of 16.10.18

Application for the assessment of ethical and legal issues of a medical research project involving human subjects: protocol

TITLE

Acquisition and promotion of visual skills in German-speaking children with and without dyslexia by learning Chinese characters.
Visual processing and pedagogical application

APPLICANT:
Prof. Dr. med. Susanne Trauzettel-Klosinski
Tel: +49 7071-298-0818, Fax: +49 7071-29-5164,
E-Mail: susanne.trauzettel-klosinski@uni-tuebingen.de

Research Unit:
Vision Rehabilitation Research Unit
Centre for Ophthalmology, University of Tuebingen
Elfriede-Aulhorn-Str. 7
D-72076 Tuebingen
Germany

Research group:
University Eye Clinic Tuebingen, Vision Rehabilitation Research Unit

- Prof. Dr. med. Susanne Trauzettel-Klosinski, head of Vision Rehabilitation Research Unit Centre for Ophthalmology, University of Tuebingen; leader, supervisor and coordinator of the project
- Dr.med. Dipl. inf. Stephan Küster, research assistant
- Angelika Cordey, orthoptist
- Theda Faisst, doctoral student
- Maria Jose Galvez de Villalta, project management

Cooperations

1. MEG Center at the University Hospital Tuebingen: Prof. Dr. Christoph Braun
There is already a long-standing cooperation with several joint publications.
2. China Centrum Tuebingen (CCT):
Prof. Dr. Helwig Schmidt-Glintzer, Director of the CCT and president of the Erich-Paulun-Institute, University of Tuebingen, Senior Professor
Utilizing a network of culture, business, academia and politics, the institute targets high school and college students who are learning the Chinese language. Dr. Vera Schick
CCT, Erich-Paulun-Institute, head of the EPI-Program, State Seminar for Didactics and Teacher Education (Grammar Schools) Tuebingen, subject leader Chinese
3. Seminar for Linguistics University of Tuebingen:
Prof. Dr. H. Baayen, head of the research group „Quantitative Linguistics“ Dr. Chin-Chu Sun (Post-doctoral researcher and native Chinese speaker)
4.Geschwister Scholl Gemeinschaftsschule Tuebingen (Rector C. Schnittert) for recruiting the normal-reading children and a part of the dyslexic ones.

Application type: Revised version
Ethics votes on previous projects are available for all investigational methods used here.
For the clinical investigation methods: 325/2009B01.
Eye Tracking in children: 227/2014BO1, for SLO-and MEG-studies 252/2007BO1.

FINANCING:
The project is funded by the Excellence Initiative of the University of Tuebingen.
In addition, the project is supported by the Charlotte and Tistou Kerstan Foundation and the Werner Kossmann Foundation.

Table of contents:

1. summary
2. introduction
    2.1 state of research
    2.2 own preliminary work
3. study objectives
4. study population
    4.1. rationale for conducting the study in minors
    4.2. recruitment
    4.3. inclusion and exclusion criteria
    4.4. study medication
5. study procedure and research methods
    5.1. study design
    5.2. conduct
    5.3. time schedule
    5.4. examination methods
6. discontinuation criteria
7. risks and side effects- clinical and scientific evaluation
8. information on statistical evaluation
9. clinical and scientific evaluation
10. insurance
11. data protection
12. education of the study participants
13. cooperation
14. literature
15. attachments

1. SUMMARY

The project is innovative in its research question and has the following foci:
A basic science-oriented part on visual and cortical processing of Chinese characters, and an application-oriented linguistic and pedagogical part of social relevance.
It is thus an interdisciplinary project and includes the following specialties: Ophthalmology, Neuro-Ophthalmology, Neuro-Sensory Science, Sinology, Linguistics, Pedagogy.

Dyslexia is a common developmental disorder of reading and writing (4-5% of German school children). Those affected are severely disadvantaged at school and in professional life, often resulting in psychological and social sequelae.
Research to date has mainly examined the children's deficits, while little attention has been paid to their strengths, especially their visual abilities.
In our research, German-speaking dyslexic children were found to have problems only with the written language stimuli, whereas pictorial material (pictograms) was processed equally well as in non-dyslexic children. Therefore, it will be investigated for the first time whether German-speaking dyslexic children can learn the non-letter logographic Chinese script as well as their normal-reading classmates, and how the characters are processed visually.
Twenty-four children each from 4th and 5th grade with and without dyslexia will be included in the study, 3 each in the pilot study.
A detailed analysis of the fixations on the characters by means of a scanning laser ophthalmoscope, which allows the reading process to be recorded directly on the retina, should provide important information about the type of processing (local or global). Magnetoencephalography will also be used to examine cortical processing.
The children will then receive a 24-hour block of Chinese lessons, for which the teaching material will be developed in a child-friendly manner.
It will be of interest to see how characters are learned in both groups and whether the dyslexics benefit.
If this were the case, dyslexic children could gain an advantage in school and their working life through the special language skills and would not have to be the "eternal losers" as they have been up to now. It could also have a positive impact on their self-confidence and their psychological and social development.
If normal-reading children are shown to be able to learn Chinese characters in 4th and 5th grade, this could lead to recommendations for Chinese language instruction in secondary schools.
In view of the increasing importance of economic and cultural relations with China, this competence would also be of educational and social benefit.

2. INTRODUCTION

2.1. state of research

Dyslexia is a circumscribed developmental disorder of reading and writing. Approximately 4-5% of pupils in German-speaking countries are affected by LRS. Despite supporting measures, the children are severely impaired in their school and professional careers and often develop psychological and social sequelae because they always feel like losers. About 30% of dyslexics become delinquent. (For

reviews see Mayer 2016, Schulte-Körne 2007, Warnke et al 2002). A generally accepted origin of dyslexia in alphabetic languages is based on the difficulty to convert letters into sounds (phonological deficit). In our own research, we were able to show that dyslexic children had difficulties only in letter-based tasks, while in naming pictograms (without time pressure) they were as good as non-dyslexic children (Trauzettel-Klosinski et al 2002).
While research to date has focused predominantly on the deficits of these children, little has been studied on their strengths and how to promote them. The potential visual talent of these children has not received sufficient attention.
An intensive field of research on logographic languages has developed only in recent years. Recent research has shown that logographic scripts such as Chinese are primarily decoded visually (Hua et al 2006, Wang LC 2016). Therefore, dyslexia in Chinese children is predominantly based on a deficit in visual processing of characters (Zhao et al 2014, Quian et al 2015 and 2016, Liu et al 2012, Meng et al 2011 and 2014, Wang et al 2010). Reports of phonological deficits in Chinese dyslexics (Cao et al 2017, Siok et al 2009) could possibly indicate subgroups with different or combined deficits.
Processing of stimuli (letters versus pictograms) in the brain occurs in different brain areas, as we found in our own research (Trauzettel-Klosinski et al 2006) (see below).
On the processing of Chinese compared to alphabetic writing in Chinese readers without reading disorder, there are some studies with functional magnetic resonance imaging (fMRI) and event-related potentials (ERPs) that have described a partial correspondence of activated brain areas but also additional activities, especially in the right hemisphere of the brain where visuospatial processing takes place (Hsu et al 2011, Wu et al 2012). In normal-reading Chinese subjects, there have also been effects of visual complexity (Hsu et al 2011), frequency of a character (Kuo et al 2003, Lee et al 2004), and orthographic consistency (Lee et al 2004) and homophonic density (Chen et al 2016) have been described.

2.2 own preliminary work

<u>Measurement of eye movements during reading</u>
A scanning laser ophthalmoscope (SLO) can be used to simultaneously image the retina and the stimuli (text or pictograms) and to view the scanning of the lines "live" on the retina (Figure 1).
When registering eye movements during reading, dyslexics make numerous gaze jumps in the direction of reading (saccades) and backwards (regressions) and have a severely slowed reading speed (Mackeben et al 2004, Trauzettel-Klosinski et al 2010). Furthermore, we have shown that reading problems increase with increasing phonological difficulty in German in dyslexic children (Dürrwächer et al 2010). Thus, eye movements can serve as an indicator of phonological deficit.

Furthermore, using magnetic encephalographic (MEG) studies, we have shown that dyslexic children had no advantage in learning a new alphabetic (Greek) script compared to a normal-reading control group due to the fact that both groups started at "zero", but were just as impaired as with the Latin script (Braun et al 2009). Thus, in our own studied collectives, we have demonstrated the phonological deficit of German-speaking readers in alphabetic language using different methods.

In contrast, the dyslexic children were able to name pictograms as quickly as normally reading children (Trauzettel-Klosinski et al 2002 b, Figure 1). The task we used consisted of naming without time pressure and without serial presentation of the pictorial stimuli. It thus differs from a "rapid naming

(RAN)" task, in which deficits have been described in the literature (Denkla & Rudel 1976, Swan & Goswami 1997, Wolf & Obregon 1992)

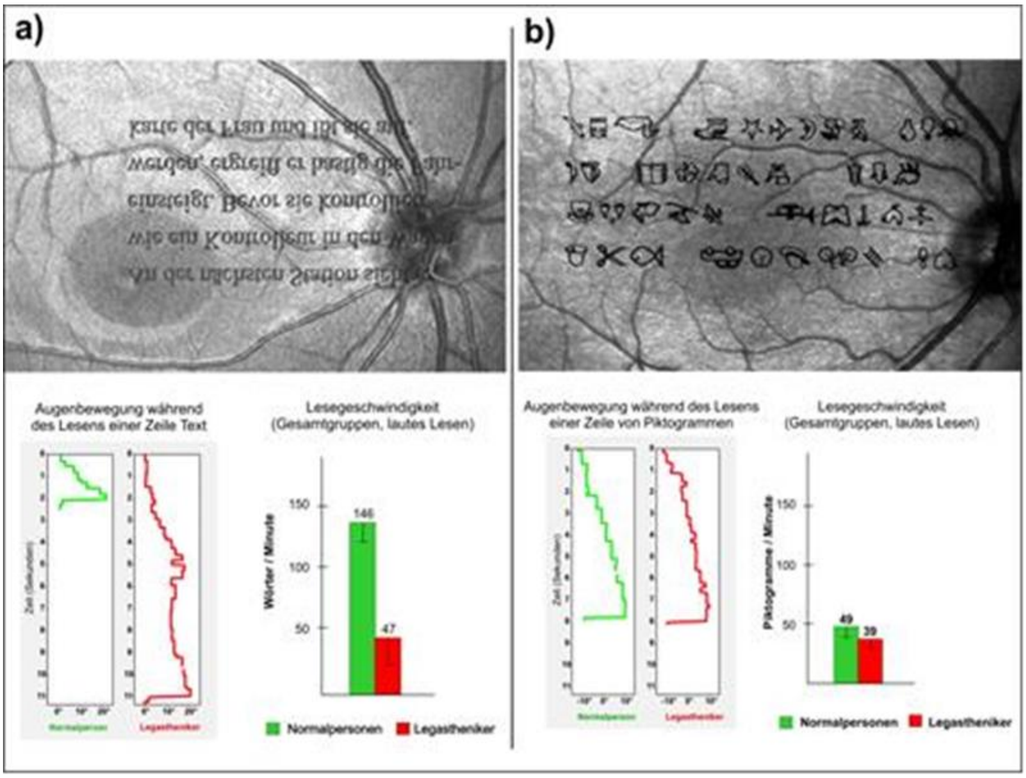

Fig 1: Measurement of the reading process by SLO: The stimuli (a: text, b: pictograms, arranged like text) are visible simultaneously with the retina. They are upside down only for the examiner, upright for the subject. a: when reading letter-bound information, the reading speed of dyslexic subjects is highly downgraded and the number of gaze jumps greatly increased. In contrast, dyslexics are not impaired when naming pictograms (b).

<u>Processing of stimuli in the brain</u>

The cortical processing of the stimuli (letters versus pictograms) takes place in different brain areas, as we found in our own studies (Trauzettel-Klosinski et al 2006). Again, dyslexics showed normal reaction times for the pictograms and prolonged reaction times for reading words (Fig 2).

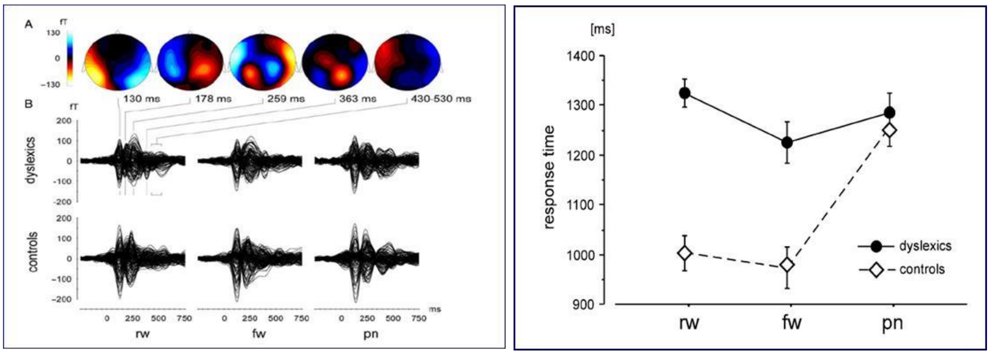

Figure 2:
Magnetic activity during the reading of words and the naming of pictograms on the Magnetoencephalogram (MEG): In the pictogram task there is no difference between the groups, while in reading words the dyslexics show delayed responses compared to the control group. rw: rare words, fw: frequent words, pn: pictograms (from Trauzettel-Klosinski et al 2006).

This led to the approach that dyslexic children might have a visual advantage if they were given pictographic/logographic rather than phonological tasks. This led to the plan that children who are dyslexic in an alphabetic language should learn a logographic language in order to possibly be as good as their classmates here.

<u>Develop standardized reading texts in 17 languages:</u>

Within the framework of an EU project (AMD-READ, QLK6-CT-2002-00214, Hahn et al 2006) and later worldwide, standardized reading texts with text sections (not only single sentences) were developed under the coordination of Prof. Trauzettel-Klosinski for the measurement of reading speed in 17 languages (Trauzettel-Klosinski et al 2012, www.precision-vision.com). There are 10 texts available for each language, which do not differ in difficulty or linguistic complexity, making them very suitable for repeated measurements. The development in the other languages followed the same principle (same content, same difficulty, same linguistic complexity) and was developed by native linguists. Chinese is also available. This makes it possible for the first time to conduct international reading studies across language boundaries.

3. QUESTIONS AND STUDY OBJECTIVES

3.1. Questions

Based on the above research results, theoretical and practical questions arise:
1) Are pictographic/logographic characters processed locally (analysis of individual components) or globally (as a whole object)? There is evidence from the literature that Chinese characters are predominantly processed globally, i.e., the character as a whole and not the individual strokes are processed.
2) Can German-speaking dyslexic children use their visual competence, which they may have already practiced as a compensatory strategy, when learning Chinese? Chinese characters are logographic and do not primarily require phonological analysis. In an earlier study (Rozin et al 1971), it was shown that dyslexic children could learn a set of 30 Chinese characters within a few hours even though they had high-level dyslexia for English. However, the children were allowed to pronounce the words in English. The question now is whether dyslexic children and adolescents could receive a boost from real Chinese language lessons that would accommodate their visual strengths and give them renewed confidence and a competitive edge in their careers.

3.2. study objectives

<u>1.visual information processing:</u>
The study of eye movements will cover the following aspects:
- Differences between logographic (Chinese) and alphabetic (German) writing.
- In the case of Chinese characters:
o Local or global processing

o Dependence on visual complexity (see Tab. 1)
o Dependence on the phonological part in the characters
This will be used to investigate whether German-speaking dyslexic children can learn Chinese characters better than alphabetic characters. The results should contribute to a better understanding of the causes of dyslexia for alphabetic writing and the associated processing disorders in the brain, as well as the possibilities of promotion via the visual system.

2.cortical information processing:
It can be assumed that the written characters are processed differently in the brain. Possibly, deficits in language areas for alphabetic reading can be compensated by good functions in language areas for logographic writing. The investigations should contribute to a better understanding of the neuronal networks underlying the processing of Chinese characters.

3.learning Chinese script and putting it into practice.
Child-friendly teaching materials are to be developed and then used and evaluated in a teaching block.

4.Educational implications:
- If the non-dyslexic children can learn Chinese as early as 4th and 5th grade, offering Chinese classes in secondary schools would be an option as the importance of Chinese language and culture will increase.
- If the dyslexic children can learn Chinese, offering Chinese classes in the schools would be an option to support these children through their visual skills (which of course should not replace the dyslexia-specific support for alphabetic language!).

5.Psychological and social impact:
Dyslexic children could, by learning Chinese, gain a
competitive advantage at school and work and would not have to be the "eternal losers" as they are now. It could also have a positive impact on their self-confidence and their psychological and social development. Consequential damage could thus be prevented.

6.Social significance:
In view of the increasing importance of cultural, economic, and scientific exchange with China, it would also be socially beneficial if more German-speaking children could learn the Chinese language.

4. STUDY POPULATION

4.1. rationale for why the study must be conducted on minors:

A reading/spelling disorder is usually diagnosed in 3rd-4th grade, sometimes as late as 5th grade. Children perceive their world primarily visually and have particularly good visual memory compared to adults, which can be an advantage for learning and remembering Chinese characters. In addition, the younger children are, the better they can learn a new language. Learning Chinese in the 4th and 5th grade, when a transition to secondary schools takes place, is a particularly favorable time for this.

4.2 recruiting

The dyslexic children are recruited via the Federal- and State Dyslexia Association (Germany and Baden-Württemberg) as well as via dyslexia diagnostic and therapy facilities in the Tuebingen area.
The recruitment of the control children is mainly done via the Geschwister Scholl Community School Tuebingen, in addition via a circular mail at the University of Tuebingen.
Special reference is made to the voluntary nature of participation.

4.3. inclusion and exclusion criteria

Inclusion criteria:

- Children with and without dyslexia in 4th and 5th grade
- confirmed diagnosis of dyslexia (with standardized test procedures)
- Willingness to attend all study appointments

Exclusion criteria

- Co-morbidities, such as ADHD
- Ocular diseases other than refractive anomalies

4.4. study medication

No drugs are tested in the study. Dilation of the pupil and cycloplegia may be necessary in some children (see Chapter 7).

5. STUDY PROCEDURE AND RESEARCH METHODS

5.1 study design

The proposed study is a prospective clinical and pedagogic diagnostic and intervention study with dyslexic and normal-reading children entering 5th grade in the fall of 2018.

Pilot study: 3 children each with and without dyslexia.
Main study: 21 children each of the 4th and 5th grade with and without dyslexia.

1) Preliminary ophthalmological examination to exclude visual deficits.
2) Examination of eye movements during reading of alphabetical text and familiar pictograms on the SLO and eye tracker.
3) Examination of the further processing of the stimuli in the brain by means of MEG
4) Block teaching of Chinese language for 2 weeks during school vacations (24 school hours, 6-8 children per learning group)
5) Control examination of eye movements while reading alphabetical text and the newly learned Chinese characters on the SLO and eye tracker.
6) Investigation of the further processing of the stimuli in the brain by means of MEG

5.2 Execution

The eye examinations are performed at the Vision Rehabilitation Research Unit
Centre for Ophthalmology, University of Tuebingen, Elfriede-Aulhorn-Str. 7,
the MEG examinations at the MEG Center of the University clinic Tuebingen, Otfried Müller Strasse.
Chinese classes are held at the China Center Tuebingen, Hintere Grabenstrasse.

5.3 Time schedule

Month 1-3: Preparation

Accurate development of the study design, development of the stimuli: selection of appropriate Chinese characters in consultation with the collaborators (newly published database, Sun 2016). Preparation of teaching materials, ethics application, recruitment of 4th and 5th grade dyslexic and normal reading children.

Month 4-6: Pilot study with 3 children each with and without dyslexia.

Testing and optimization of stimuli, testing and optimization of teaching materials and delivery of lessons. First Teaching block. Recruitment for the main study

Month 7-12: Start of main study: data collection and teaching.

Month 13-18: Continuation of data collection, teaching, start of analysis

Month 19-21: Overall evaluation of all data, very time consuming

Month 22-24: Writing of publications, public relations, transfer into practice

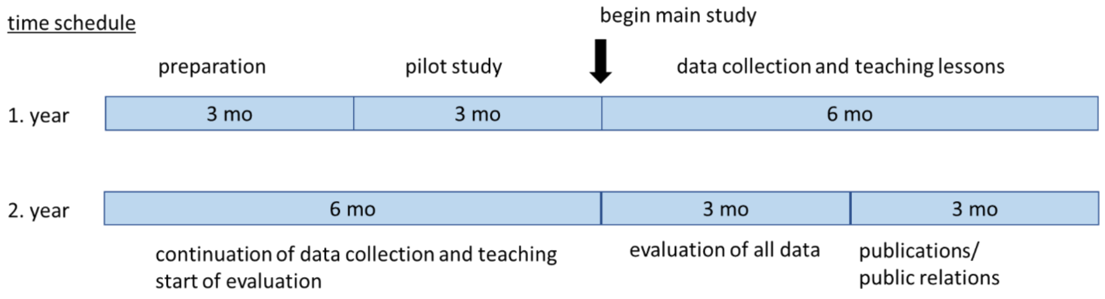

5.4. examination methods

All examinations are performed on an outpatient basis. The initial examination in the eye clinic takes about 1.5- 2 hours per patient with additional breaks. The control examination lasts 0.5 -1 hour. The MEG examinations usually take place on another day and are scheduled for about half an hour.

5.4.1. Preliminary ophthalmic-orthoptic examination

- Determination of visual acuity for distance and near at standardized illumination level. Included is the objective refraction determination by means of skiascopy or refractometer.
- Orthoptic status: fixation, eye position, motility, binocular vision, eye dominance
- Clinical-morphologic examination of the anterior and posterior segments of the eye with accurate assessment of ophthalmologic findings.

5.4.2. Quality of life questionnaires

A standardized quality of life questionnaire is used at the beginning of the study

5.4.3. Measurement of eye movements

Eye movements during the reading of German and Chinese text as well as during the naming of pictograms are measured by means of an infrared eye tracker (Saccadometer, Jazz novo, Ober, Figure 3). The measurement unit is located at the root of the nose and tangentially illuminates the limbus. It is attached to the head with an elastic band. The system thus has the advantage, especially for children, of not sitting directly in front of the eyes. We have already used the method in children in a study that has just been completed (Ethics vote: 227/2014BO1).

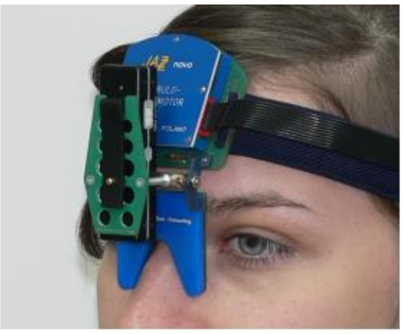

Figure 3: Infrared Limbus Eye tracker

5.4.4. 5.4.4 Study of visual processing of the stimuli (alphabetical and pictographic)

The following methods are available:

On the retina:

With a Scanning Laser Ophthalmoscope (SLO), the retina and the presented characters can be displayed simultaneously and exactly how a character is scanned can be recorded (see Fig. 1). Thus, one can see a live image of the reading process directly on the retina. With the targeted selection of Chinese characters, the following questions will be investigated by means of the fixations on the character:

- Local (fixation of individual strokes) or global (as a whole object) processing.
- Dependence on visual complexity (Table 1)
- Influence of the iconographic degree of abstraction

Table 1: Examples of low and high visual complexity (after Sun 2016). As the number of strokes increases, the character becomes more complex.

Character	Stroke	Meaning
个	3	each
露	21	expose

Cortical processing

Furthermore, the processing of stimuli in the brain can be recorded by means of magneto-encephalography MEG with high temporal resolution (milliseconds) (in cooperation with the MEG center) (see Fig. 2). As with SLO, this is a completely non-invasive method, which we have also used in previous studies in children.

5.4.5. Chinese lessons

First, the teaching material is developed by our cooperation partner at the CCT in a child-friendly way. Then the children (dyslexic and controls) receive block lessons during the school vacations: In small groups of 6-8 students, 3-hour lessons take place on each of 8 vacation days with a break. Thus, each child receives a total of 24 school hours (45 minutes each) of Chinese lessons.

6. DISCONTINUATION CRITERIA

Patients may discontinue participation in the study at any time without giving a reason.

7. RISKS AND SIDE EFFECTS

The ophthalmologic examinations performed in the study correspond to those of a routine ophthalmologic examination with regard to risks and possible side effects.
For the examination of the ocular fundus, pupil dilation with a combined parasympatholytic and sympathomimetic drug is occasionally required. For pupil dilation, we use the parasympatholytic drug tropicamide topically, which is also used in routine ophthalmologic examinations. There is little risk of glaucoma due to pupil dilation in children.
For objective refraction determination, cycloplegia is sometimes required in younger children. For this purpose, we administer eye drops containing the active ingredient cyclopentolate hydrochloride (cyclopentolate 1%, or 0.5%), which are usually dripped 2-3 times at intervals of 10 minutes.
The pupil dilation lasts for about 3-4 hours. During this time, there may be an increased sensitivity to light and a reduction in near vision.
The infrared eye tracker used here is particularly suitable for children, as it is only attached with a headband and the measuring unit does not sit directly in front of the eyes.

The Scanning Laser Ophthalmoscope (SLO) scans the retina with a weak laser beam. The device has two independently operating safety systems and meets the requirements of the weakest laser safety class I, which means that no harmful laser radiation can affect your eye at any time. The laser has a purely diagnostic function.
The SLO allows simultaneous acquisition of the retinal image and the stimulus and therefore provides the absolute position of the fovea on the stimulus. It requires pupil dilation in most patients. The method has been used by us for many years for reading analysis (ethics votes 077/2012 BO1, 176/2003V).

8. CLINICAL AND SCIENTIFIC EVALUATION

The study will provide important theoretical insights into the information processing of letter-based versus pictographic (logographic) writing. For the processing of Chinese characters, we will obtain important information regarding local or global processing as well as dependence on visual complexity and phonological content.
The application consists in concrete Chinese language teaching lessons. If the normal-reading children can learn the Chinese characters, such an offer in the secondary schools would be an option. For the dyslexic children, it would offer the opportunity to use their visual strengths and to have a positive record in their school and also professional careers through these language skills. This would also be of social relevance, considering the increasing importance of economic, cultural, and scientific relations with China.

9. INFORMATION ON THE STATISTICAL EVALUATION

It is planned to analyze the data by means of an analysis of variance and to use non-parametric tests in case of non-normal distribution.

10. insurance

For the way to or from the examination appointments as well as for the lessons, a travel and accommodation insurance is taken out. Travel accident insurance no. 50073896666, Insurer: SV SparkassenVersicherung AG

11. data privacy

The data are collected on questionnaires or examination forms and stored in computer files. The data are stored in pseudonymized form immediately after collection. For this purpose, a code is assigned, and a list is kept that would allow the data to be traced.

The coding records and the coding list, together with the consent forms, are kept in a locked cabinet to which only the investigators have access. It will be made available to third parties only in the cases specified in the privacy consent form. The data will be kept for 10 years.
Separate information sheet of the University clinic Tuebingen will be enclosed.

12. Education of the study participants

Each subject and each patient with at least one parent will be informed in detail about the procedure of the study by the investigator before they give their consent. All subjects/patients and their parents are explicitly informed that they can terminate their participation, or the participation of their child, in the study at any time and without giving reasons. Only then do the study participants and their parents give their consent to the studies.
Compensation: The children will receive a voucher (books, visit to the cinema) worth 25 € after completion of the last examination, the parents will also receive an expense allowance of 25 €.

13. Cooperation (see page 1 for details)

Tuebingen, October 16th, 2018

Prof. Dr. med. Susanne Trauzettel-Klosinski

15. attachments
information and consent sheet
